# A semiochemical view of the ecology of the seed beetle *Acanthoscelides obtectus* Say (Coleoptera: Chrysomelidae, Bruchinae)

**DOI:** 10.1111/aab.12862

**Published:** 2023-09-04

**Authors:** József Vuts, Stephen J. Powers, Eudri Venter, Árpád Szentesi

**Affiliations:** ^1^ Protecting Crops and the Environment Department Rothamsted Research Harpenden UK; ^2^ Stats Powers Ltd Somerset UK; ^3^ Rothamsted Bioimaging Rothamsted Research Harpenden UK; ^4^ JEOL UK Welwyn Garden City UK; ^5^ Department of Systematic Zoology and Ecology Eötvös Loránd University Budapest Hungary

**Keywords:** Bruchinae, Chrysomelidae, Coleoptera, IPM, Leguminosae, oviposition, plant–insect interaction, semiochemical

## Abstract

The dried bean beetle, *Acanthoscelides obtectus*, is an economically important pest of stored legumes worldwide. Tracking the human‐aided dispersion of its primary hosts, the *Phaseolus vulgaris* beans, it is now widespread in most bean‐growing areas of the tropics and subtropics. In temperate regions where it can only occasionally overwinter in the field, *A. obtectus* proliferates in granaries, having multiple generations a year. Despite its negative impact on food production, no sensitive detection or monitoring tools exist, and the reduction of local populations still relies primarily on inorganic insecticides as fumigating agents. However, in the quest to produce more nutritious food more sustainably and healthily, the development of environmentally benign crop protection methods is vital against *A. obtectus*. For this, knowledge of the biology and chemistry of both the host plant and its herbivore will underpin the development of, among others, chemical ecology‐based approaches to form an essential part of the toolkit of integrated bruchid management. We review the semiochemistry of the mate‐ and host‐finding behaviour of *A. obtectus* and provide new information about the effect of seed chemistry on the sensory and behavioural ecology of host acceptance and larval development.

## Graphical abstract

The dried bean beetle, *Acanthoscelides obtectus*, is an economically important pest of stored legumes worldwide. We review the semiochemistry of the mate‐ and host‐finding behaviour of *A. obtectus* and provide new information about the effect of seed chemistry on the sensory and behavioural ecology of host acceptance and larval development. Knowledge of the biology and chemistry of both the host plant and its herbivore will underpin the development of chemical ecology‐based approaches to form an essential part of the toolkit of integrated bruchid management.

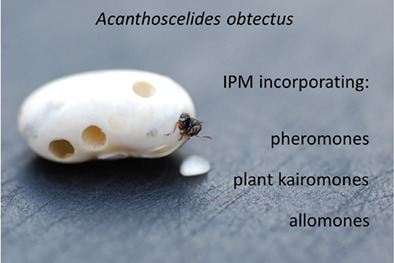

## 1 INTRODUCTION

*Acanthoscelides obtectus* Say (Coleoptera: Chrysomelidae, Bruchinae) is a worldwide pest of dry beans, *Phaseolus vulgaris* L. (Leguminosae; Alvarez et al., 2005). Females scatter their eggs into drying pods in the field and leg‐bearing first instar larvae actively choose and bore into the seeds, which are then harvested and serve as sources of infestation in store houses for multiple generations of the beetle (Tuda, 2007). Its ability to use dry, hard beans, similar to other bruchids in the *Callosobruchus* and *Zabrotes* genera, is proposed by Tuda et al. (2006) to originate from a preadaptation that precedes human storage of legumes that has been selected for by arid habitat climate. It is thought that *A. obtectus* represents an early stage of bruchid evolution because of the anatomy of its first instar larvae and its capability to infest crops both pre‐ and post‐harvest (Parsons & Credland, 2003), and it is suggested by Alvarez et al. (2005) that the multi‐generation character of the genus is ancient (plesiomorph), not derived (apomorph). The control of *A. obtectus* using various chemical, biological, mechanical and cultural methods has met with varied success (Abate & Ampofo, 1996; Boyer et al., 2012; Mutungi et al., 2015; Velten et al., 2008; Yankova & Sofkova, 2013), and sensitive and specific detection and monitoring approaches are still required. Similar to other stored product pest insects, semiochemical‐based management strategies may provide environmentally benign tools for surveillance and direct reduction of local *A. obtectus* populations (Trematerra, 2012). We give an overview of the semiochemistry of *A. obtectus*, with the aim to create a platform for new studies developing novel management programmes.

Semiochemicals (behaviour‐ and development‐modifying chemical signals) can be divided into two broad groups based on the taxonomic relation of the participants: pheromones are used for intraspecific communication (Karlson & Lüscher, 1959), whereas allelochemicals convey information in an interspecific context (Whittaker & Feeny, 1971). The semiochemistry of bruchids has been reviewed previously (Francke & Dettner, 2005; Rodríguez, 2018). The reviews highlight the widespread occurrence of female‐produced sex pheromones in the subfamily. Information on bruchid allelochemicals (e.g., host attractants, arrestants, repellents and natural products affecting larval development), however, is sparse. Sensilla on the antennae and palpi (Urbanek et al., 2016), and likely on the ovipositor, are proposed to have chemosensory functions for both pheromone and host plant compounds, but functional annotation studies are yet to be conducted.

## 2 CHEMICAL ECOLOGY IN STORE HOUSE ENVIRONMENTS

*Acanthoscelides obtectus* originates in the Neotropics, but it has become cosmopolitan through human‐mediated migrations since the domestication and distribution of beans (Alvarez et al., 2005). It can have several generations a year in the tropics and subtropics, depending on climatic and ecological conditions (Huignard & Biemont, 1978), which characteristic is thought to enable it to reproduce continuously under storage conditions in temperate regions. With a virtually constant supply of legume seeds available, overlapping generations can exist and intraspecific chemical communication predominates.

### 2.1 Mate finding

**FIGURE 1:**
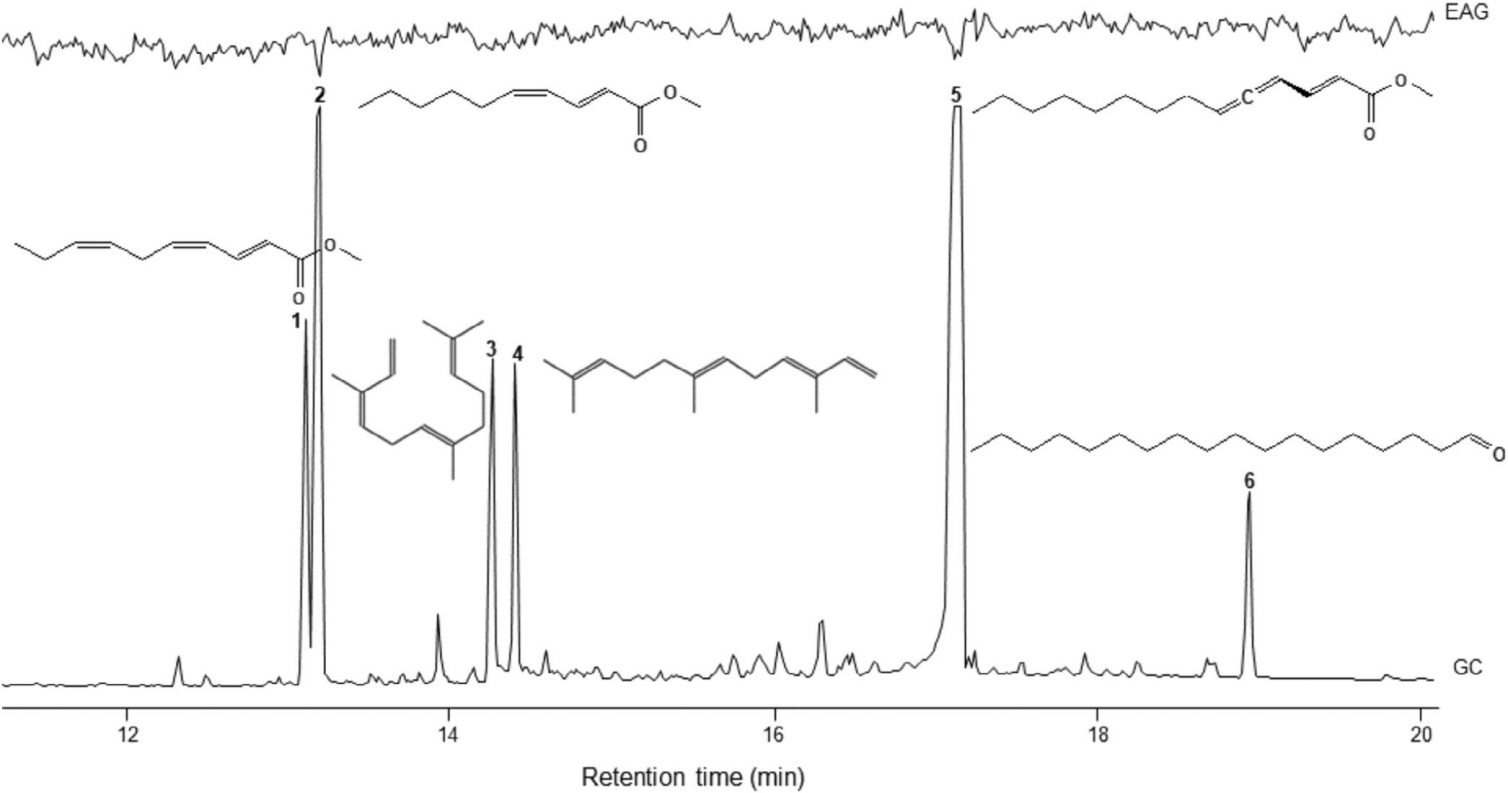
Antennal responses of virgin female *Acanthoscelides obtectus* to constituents of a male air entrainment extract in GC–EAG. 1: methyl (2*E*,4*Z*,7*Z*)‐2,4,7‐decatrienoate, 2: methyl (2*E*,4*Z*)‐2,4‐decadienoate, 3: (3*Z*,6*E*)‐α‐farnesene, 4: (3*E*,6*E*)‐α‐farnesene, 5: methyl (*E*,*R*)‐2,4,5‐tetradecatrienoate, 6: octadecanal. Only compounds 2 and 5 evoked reproducible EAG responses (*n* = 4). Antennal recordings were made using Ag–AgCl glass electrodes filled with saline solution composed as in Maddrell (1969), but without the glucose. An antenna was freshly amputated at the base from a live *A*. *obtectus* and suspended between the two electrodes. The tip of the terminal process of the antenna was removed to ensure a good contact with a high‐impedance amplifier (Ockenfels Syntech GmbH, Kirchzarten, Germany). The base of the antenna was connected to a grounded electrode. Separation of the collected male *A. obtectus* volatiles was achieved on a high‐resolution gas chromatograph, equipped with a cool on‐column injector, a flame ionisation detector (FID) and a non‐polar column. One microlitre aliquots of headspace extract were injected into the GC. The outputs from the EAG amplifier and the FID were monitored simultaneously and analysed using a customised software package (Ockenfels Syntech GmbH, Kirchzarten, Germany). See Wadhams (1990) and Vuts, Woodcock, König, et al. (2018), as well as Suppl_1, for detailed methodology.

Sex pheromones guide the receiver to the emitter of the opposite sex for mating and are typically multi‐component mixtures (Wyatt, 2017). *A. obtectus* has a male‐produced sex pheromone, which makes it unique among other bruchids, where typically the female is the producing sex. (*Bruchus rufimanus* Boheman, an unrelated but also economically important bruchid, may be another exception, where the presence of a male‐emitted sex pheromone compound, 1‐undecene, has been suggested; Bruce et al., 2011.) Hope et al. (1967) first reported a single sex‐specific compound isolated from hexane surface extracts of *A. obtectus* males and proposed it either to stimulate the emergence of females or to be a sex attractant. This compound was later identified as the allenic methyl (2*E*)‐2,4,5‐tetradecatrienoate (Horler, 1970), and it was shown to have the (4*R*)‐configuration (Pirkle & Boeder, 1978). Horler (1970) and Halstead (1973) noted that sections of thin layer chromatograms containing the allenic ester did not consistently evoke attraction from females. Horler (1970) thus proposed that the attractant is a similar chemical usually present in these fractions and that there are at least two other closely related bioactive compounds. Octadecanal was later identified in solvent extracts of males and found to synergize the activity of the ester as an attractant for females (Annoscia et al., 2010). In addition, unspecified C16 and C18 methyl and ethyl esters were reported from solvent extracts of both sexes, along with a stereochemically undefined α‐farnesene (Gołębiowski et al., 2008). Initial studies by Francke and Dettner (2005) suggested dynamic headspace collection to be more efficient than solvent extraction to obtain sex‐specific volatiles from male *A. obtectus*. Vuts, Powers, et al. (2015) applied this technique to collect samples from unmated males and identified methyl (*E*,*R*)‐2,4,5‐tetradecatrienoate, methyl (2*E*,4*Z*,7*Z*)‐2,4,7‐decatrienoate, methyl (2*E*,4*Z*)‐2,4‐decadienoate, octadecanal and the sesquiterpenes (3*Z*,6*E*)‐ and (3*E*,6*E*)‐α‐farnesene to be consistently present in the aeration extracts. None of these compounds were found in samples from females. Coupled gas chromatography–electroantennography (GC‐EAG) tests with female antennae assigned bioactivity to only two of the six male‐specific components (Figure 1).

However, after obtaining authentic standards (Mori, 2015), four‐arm olfactometer bioassays with synthetic blends mimicking the amount and composition of pheromone emitted by a single beetle revealed that all six were necessary for full female behavioural activity (‘attraction’; Vuts, Powers, et al., 2015). Interestingly, as opposed to Halstead (1973), gravid females also showed preference for the synthetic male pheromone blend in our olfactometer tests (Table 1). In agreement with Halstead (1973), we could not show male preference for the synthetic male pheromone (Table 1). These studies also highlight that behavioural tests assess the biological activity of a compound more sensitively than antennal electrophysiology, and that the latter technique does not indicate what type of behaviour an electrophysiologically active compound elicits.

**TABLE 1:** Behavioural responses (mean time spent searching [min] ± SE) of *Acanthoscelides obtectus* individuals to the synthetic male pheromone blend in four‐arm olfactometer assays (*n* = 10).

Beetle	Male pheromone	Control	*p*‐value
Mated female	2.91 ± 0.60	1.61 ± 0.28	.028
Virgin male	2.52 ± 0.89	2.33 ± 0.51	.852
Mated male	2.29 ± 0.71	2.45 ± 0.41	.850

*Note*: Glass arms were attached to the end of each of the four arms. The olfactometer was illuminated from above by diffuse uniform lighting screened with red acetate and was surrounded by black paper to remove any external visual stimuli. Test compounds were applied onto filter paper strips in proportions and doses in such a way that the amounts released per hour were similar to those emitted by one male beetle over 1 h. One treated and three control arms were used, thereby ensuring the robustness of the experiment by making it less likely for an insect to accidentally walk in or out of the treated region. A single beetle was introduced through a hole in the top of the olfactometer. Air was drawn through the central hole by a vacuum pump and, consequently, pulled through each of the four side arms. Each experiment was run for 16 min. The olfactometer was rotated 90° every 4 min to control for any directional bias. The olfactometer was divided into four regions, corresponding to each of the four arms, and the time spent in each arm was recorded. A linear mixed model (LMM), fitted using the method of residual maximum likelihood (REML), was applied to the data, which takes account of the design structure of olfactometer replicate runs and areas within them (as split‐plots) before testing (*p <* .05, approximate *F*‐test), followed by Fisher's LSD test. Genstat (18th Edition, VSN International Ltd, Hemel Hempstead, UK) was used for this analysis. See Pettersson (1970) and Vuts, Woodcock, König, et al. (2018), as well as Suppl_1, for detailed methodology.

Although adult bruchids often feed on nectar to gain energy for sustained flight (Clement, 1992) and on pollen (Szentesi, 2006), most of the precursors for pheromone biosynthesis in *A. obtectus* are thought to be accumulated during larval development. The hypothesis that nutritional composition of the seed cotyledon determines which precursors are available in the adult stage for de novo pheromone production was validated by Vuts, Woodcock, König, et al. (2018), who reared beetles on seeds of both the ancestral host *P. vulgaris* and of the acceptable non‐host, chickpea (*Cicer arietinum* L.). Intriguingly, there was an almost complete lack of methyl (2*E*,4*Z*,7*Z*)‐2,4,7‐decatrienoate in the headspace extract of 1st generation males reared on chickpea. However, the emission of this compound returned after rearing 1st generation chickpea beetles on bean seeds again. The biosynthesis of methyl (2*E*,4*Z*,7*Z*)‐2,4,7‐decatrienoic acid, that is, the acid part of the ester, was postulated to be rationalised either via lipoxygenase‐mediated cleavage of (9*Z*,12*Z*,15*Z*)‐9,12,15‐octadecatrienoic acid (α‐linolenic acid) or by a sequence of four β‐oxidation steps and rearrangement of the same precursor (Vuts, Powers, et al., 2015). In addition, the amount of methyl (*E*,*R*)‐2,4,5‐tetradecatrienoate, the most abundant constituent of the male sex pheromone, doubled after the bean–chickpea–bean transitions. As bean seeds contain five times more α‐linolenic acid than chickpea seeds (Grela et al., 2017), this creates a platform for new hypotheses to be formed about the biosynthetic origins of these compounds. Interestingly, females from the two host lines responded differently to male chemical signals (Vuts, Woodcock, König, et al., 2018), the bean‐reared females not differentiating between the bean and chickpea male pheromone blends, possibly reflecting a broad acceptance range of sex pheromone composition, that is a high degree of behavioural phenotypic plasticity. In contrast, females reared on chickpea showed preference for the male pheromone blend of their own host line, even in the first generation, indicating a high excitatory state of the central nervous system, causing increased reactivity to their own host line blend (sensitisation). However, EAG responses to male odour using antennae of female host lines were similar, all preferring bean‐reared males, and egg‐laying choice tests revealed a uniform preference for bean seeds across female host lines, even after multiple generations. Vuts, Woodcock, König, et al. (2018) thus concluded that the development of divergent chemical signalling systems during host shifts does not facilitate the evolution of host races in *A*. *obtectus*, because oviposition preferences remain unaffected.

### 2.2 Mate recognition and anti‐aphrodisiac functions

A constituent of the male *A. obtectus* sex pheromone, the allenic ester methyl (*E*,*R*)‐2,4,5‐tetradecatrienoate, has other functions in the chemical communication of the species. Male beetles in search of mating partners actively tap the dorsal surface of conspecifics and initiate copulation upon contact with a female, but not another male; thus, the role of cuticular hydrocarbons in mate recognition has been suggested (Stojković et al., 2014). Analysis of solvent extracts of males and virgin females revealed very similar cuticular hydrocarbon profiles, apart from methyl (*E*,*R*)‐2,4,5‐tetradecatrienoate and octadecanal characterising male extracts only (Vuts, Powers, et al., 2015). A series of choice assays demonstrated that the presence of the methyl ester serves contact mate recognition, signalling that the encountered individual is a male. By agitating male body parts in dry silica gel and extracting the gel with organic solvent, Vuts, Francke, et al. (2015) revealed that the allenic ester is part of the wax layer of the epicuticle (approximately 1 μg/male in total) and is most abundant on the thorax and elytra (37.5% and 39.1%, respectively), as also reported by Hope et al. (1967). However, the lower molecular weight components of the male sex pheromone, that is the C10 methyl esters and the two α‐farnesenes, were only detected in trace amounts in surface or direct solvent extracts of freeze‐killed individuals, compared to aeration extracts from live beetles (Vuts, Powers, et al., 2015), indicating that these compounds are produced de novo. Biemont et al. (1990) suggest that methyl (*E*,*R*)‐2,4,5‐tetradecatrienoate is emitted by ampullate pygidial glands, which implies that the secretion is transferred from the pygidium to the other body parts physically (smeared across), given the relatively low abundance of the compound on the pygidial surface (6.4%). The anatomical origin of the other pheromone constituents remains unknown, although Biemont et al. (1990) identified other glandular structures on abdominal tergites as possible sources.

Interestingly, methyl (*E*,*R*)‐2,4,5‐tetradecatrienoate is also utilised by *A. obtectus* as an anti‐aphrodisiac. Males transfer this compound onto females presumably via physical contact during copulation (the ester is present on the abdominal sternites in 13.6% of total extractable amount), rendering them unattractive for other males for up to 2 days (Huignard, 1974; Vuts, Powers, et al., 2015). This may be a fitness advantage for females, which suffer less male harassment up until the chemical signal erodes below a threshold level, at which point they are no longer recognised as a mated individual.

These findings underline the parsimonious use of the same compound in different intraspecific chemical communication channels (Blum, 1996). Johansson and Jones (2007) suggest that signals used in species recognition could evolve from signals with mate recognition, or mate assessment, functions. Some of these signals are predicted not to be under high selective pressure to evolve species‐specificity (Brent & Byers, 2011) and can comprise ubiquitous chemicals active in a number of biological systems. In light of this, male‐produced methyl (*E*,*R*)‐2,4,5‐tetradecatrienoate may have been utilised originally only for mate recognition in *A. obtectus*, but a new role as regards species recognition has emerged.

**FIGURE 2:**
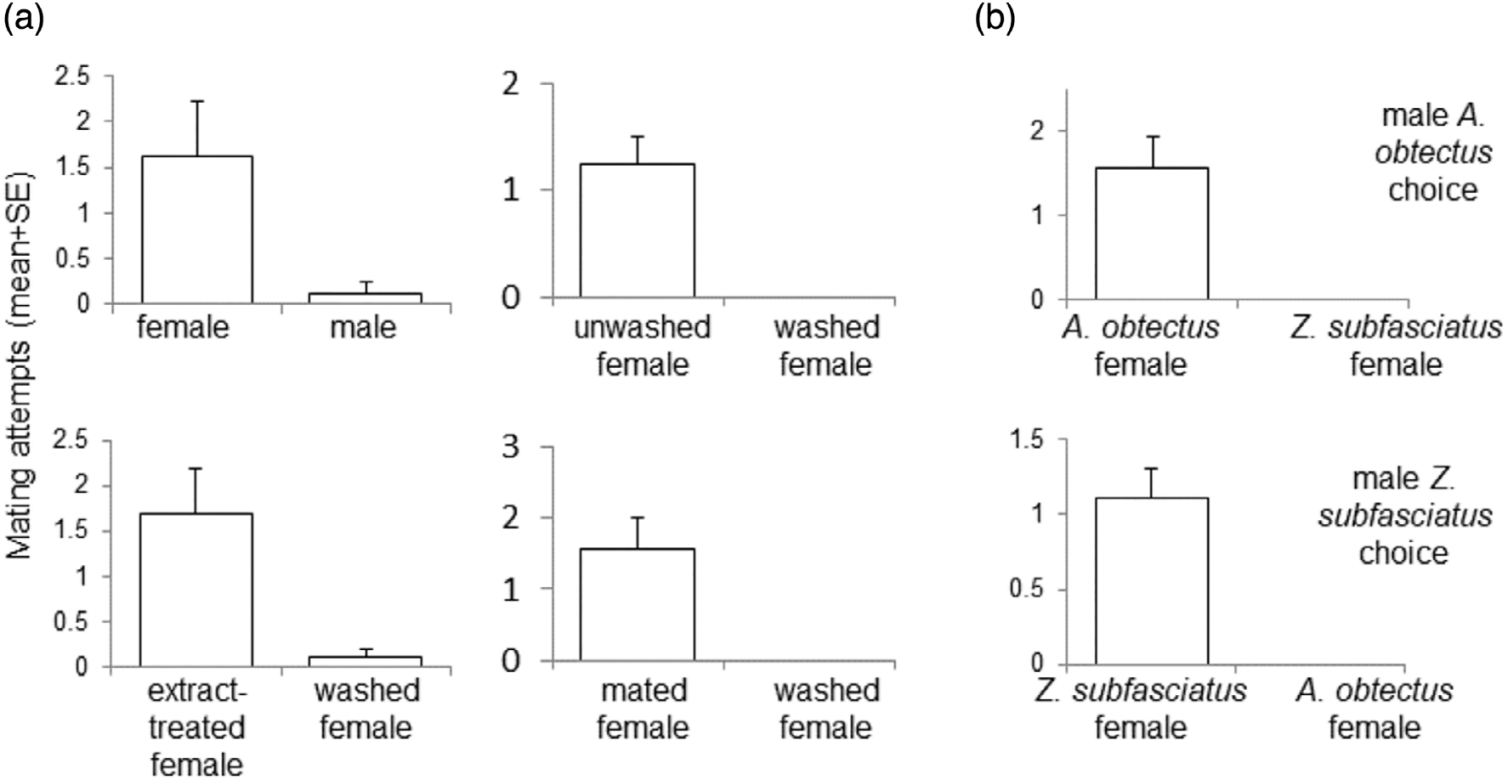
Results of Petri dish arena choice assays. (a) male *Zabrotes subfasciatus* choices between conspecific dummies. (b) male *Acanthoscelides obtectus* and *Z. subfasciatus* choices between female dummies of both species. Females were freeze‐killed on dry ice before use in experiments and were laid on their side. If a beetle was soaked in hexane, the solvent was allowed to evaporate prior to testing. One male beetle was put in each Petri dish arena (representing 1 replication, *n* = 7–10), and the number of copulation attempts (mounting and penis extruded) towards the test and control freeze‐killed individuals in 20 min was recorded. A generalised linear model was applied to the count data. See Vuts, Francke, et al. (2015) and Suppl_1 for detailed methodology.

*Zabrotes subfasciatus* Boheman, that co‐occurs with *A. obtectus* in store house environments without negative effect on each other's population dynamics (Mallqui et al., 2013), can also distinguish between the sexes by contact chemoreception (J. Vuts et al., unpublished). Males readily recognise freeze‐killed conspecific virgin females, mount them and attempt copulation (Figure 2a). The stimulatory cuticular signals can be removed by organic solvent, washed females thus losing their activity, but regaining it after treatment with the female solvent extract. Mated females evoke similar activity by males as virgin ones. The tests also revealed that males of each bruchid species prefer to initiate mating with conspecific females (Figure 2b). The question of which chemical signals on the cuticular surface confer species recognition remains open.

### 2.3 Egg‐laying and host‐marking

Several insect species utilise host‐marking pheromones to reduce the negative consequences of intraspecific competition on their offspring, thereby increasing the fitness of the marking individual (Nufio & Papaj, 2001). Such deposited chemical markers signal to conspecific females that a given host is occupied, which is thus avoided for egg‐laying. Most known host‐marking pheromones have low volatility, are deposited during egg‐laying (Hilker & Meiners, 2002) and are detected by contact chemoreception; contact with them has been shown to promote dispersal by both females and offspring away from a host or patch of hosts. The presence of host‐marking pheromones has been shown in *Callosobruchus* species (Sakai et al., 1986) and their composition identified in *Callosobruchus chinensis* L. as a mixture of saturated hydrocarbons and diacylglycerols (Kumazaki et al., 2000). Szentesi (1981) described the presence of a yet unknown substance that results in avoidance of marked beans by other *A. obtectus* females for oviposition. Also, the marking pheromone increases the length of larval wandering periods before host seeds are entered, thereby enabling larvae to find relatively less exploited host patches. No specific marking behaviour by adults was noted; thus, it is thought that the marking substance is incidentally left by adult *A. obtectus* females and males during defecation. Interestingly, extracts made from seeds defecated on by males had a stronger effect on seed avoidance by females than female extracts, underlining the possible origin of the marking substance in the faeces. Szentesi (1981) showed it to be extractable by both polar and apolar solvents from the seed surface and suggested it to be composed mostly of fatty acid derivatives. A study by Nazzi et al. (2008) indicates that C27–28 hydrocarbons may also have a role in the repellent effect of already occupied bean seeds visited by adult beetles. Parsons and Credland (2003) emphasise the importance of the presence of exit holes in adult avoidance of infested beans.

Chemistry of the seed coat (testa) also affects female oviposition site choice, with surface compounds acting as cues that govern host seed recognition. The bean seed testa contains polyphenols, such as tannins, which are trypsin inhibitors that insect herbivores need to overcome. Red and black bean cultivars contain higher levels of polyphenols than white cultivars (Fernández et al., 1982), which may confer them stronger resistance against seed predators. Such resistance mechanisms involve the impact on the ability of L1 larvae to enter and develop within the seed (see below) and oviposition deterrence by contact chemical cues. Paired choice experiments with bean seeds surface‐treated with specialised (‘secondary’) plant metabolites (SPMs) of legumes revealed that the tested compounds elicited oviposition deterrence at various degrees (Á. Szentesi, unpublished). In particular, the lowest number of eggs were laid on tannin‐treated beans, followed by morin, brucine and cis‐aconitic acid, and even some carbohydrates (Table 2).

**TABLE 2:** Deterrence ability of various organic compounds on egg‐laying of *Acanthoscelides obtectus* in binary choice‐tests comparing surface‐treated and control bean seeds.

Class and compound	Control mean	Treated mean	Mean of differences	SE	*t*‐statistic	df	*p*‐value
Organic acids							
Oxalic acid	310.7	76.1	234.6	34.78	6.74	6	<0.001
Nicotinic acid	415.1	62.7	352.4	32.10	10.98	6	<0.001
cis‐Aconitic acid	443.0	48.0	395.0	24.07	16.41	6	<0.001
Tartaric acid	274.1	150.7	123.4	44.56	2.77	6	0.032
Fumaric acid	324.6	84.6	240.0	24.23	9.91	6	<0.001
DL‐Malic acid	306.9	129.9	177.0	33.44	5.29	6	0.002
Salicylic acid	309.6	98.7	210.9	41.28	5.11	6	0.002
Maleic acid	415.6	47.6	368.0	30.69	11.99	6	<0.001
Succinic acid	395.1	101.3	293.9	21.51	13.66	6	<0.001
Malonic acid	301.3	109.0	192.3	21.42	21.42	6	<0.001
Sodium‐citrate	249.1	238.6	10.57	45.14	0.23	6	0.823
Magnesium citrate	286.9	141.6	145.3	19.05	7.63	8	<0.001
Others							
Rutin	443.1	32.4	410.7	14.16	29.00	6	<0.001
Codeine	380.4	81.3	299.1	36.35	8.23	6	<0.001
Salicin	326.4	102.6	223.9	17.78	12.59	6	<0.001
Colchicine/0.004 M	314.0	182.9	131.1	39.88	3.29	6	0.017
Ergotamine tartrate/0.007 M	302.7	140.4	162.3	20.38	7.96	6	<0.001
Tomatine/0.086 M	337.6	102.9	234.7	18.64	12.59	6	<0.001
Morin	420.1	17.6	402.6	12.46	32.31	6	<0.001
Brucine	380.4	20.0	360.4	11.77	30.61	6	<0.001
Isatin	368.6	70.7	297.9	24.11	12.36	6	<0.001
Tannin (catechin)/2% w/v	403.6	31.7	371.9	10.19	36.49	6	<0.001
Solasodine	363.3	47.6	315.7	29.68	10.64	6	<0.001
Atropine	380.8	51.4	329.3	21.54	15.29	8	<0.001
Nicotine H‐tartrate	295.2	144.8	150.4	25.11	5.99	8	<0.001
Carbohydrates							
D‐Raffinose	244.6	198.0	46.57	16.66	2.80	6	0.031
L‐Rhamnose	275.6	242.7	32.86	24.70	1.33	6	0.232
Galactose	249.3	217.7	31.57	18.42	1.71	6	0.137
D‐Mannose	259.1	262.1	−3.00	19.06	−0.16	6	0.880
Saccharose	277.6	210.4	67.14	31.37	2.14	6	0.076
D‐Glucose	271.3	222.0	49.29	30.09	1.64	6	0.153
D‐Sorbitol	279.3	143.7	135.6	20.72	6.54	6	<0.001
L‐Arabinose	228.8	193.2	35.56	21.07	1.69	8	0.130
D‐Arabinose	240.1	203.4	36.67	23.94	1.53	8	0.164
D‐Fructose	281.1	165.9	115.2	21.41	5.38	8	<0.001
D‐Xylose	223.0	211.1	11.89	16.53	0.72	8	0.492
Dextran/0.01M	280.6	66.6	214.0	19.37	11.05	8	<0.001

*Note*: The mean total numbers of eggs laid for surface‐treated and control seeds are shown per compound, along with the mean of differences and paired *t*‐test results. A given compound and its corresponding control were compared in a 10 cm diameter glass Petri‐dish divided into four sections by a paper or glass cross stuck to the bottom to hinder mixing of treated and control beans. The same treatment was placed at the opposite sections of the dish and the orientation of dishes was randomised. Then, 10 male and 10 female beetles, 2–3 days old, were placed in a dish for 10 days with the dish being kept at 23°C in complete darkness (*n* = 7 or 9). For details, see Suppl_2.

**FIGURE 3:**
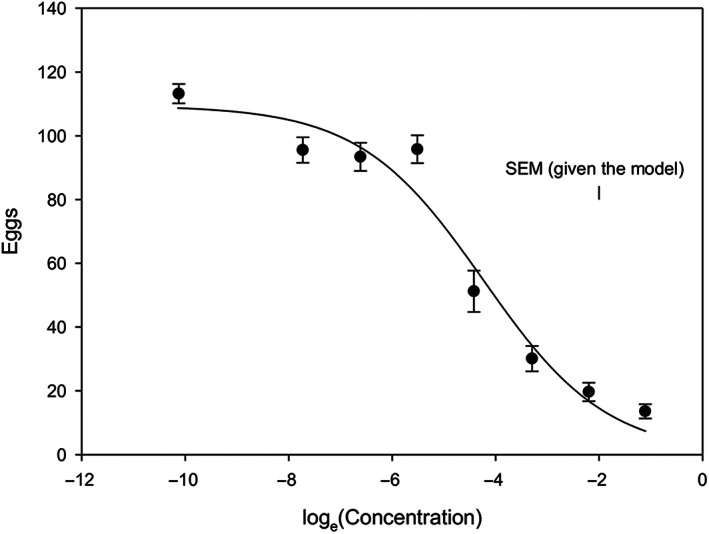
Mean number of eggs laid (±SE) at different tannin concentrations, a fitted logistic curve relationship and the standard error of the mean number of eggs laid given the model (SEM). The estimated parameters (SE) in the model, *Eggs* = *C*/(1 + exp[*B* log_e_(Concentration) – *M*]), were *B*: 0.8389 (0.0899), *C*: 109.46 (3.68) and *M*: −4.237 (0.158). The ED50, here the concentration which reduces the number of eggs laid by 50% (exp(*M*)), was 0.01441 (0.00229). Variance explained (*R* ^2^) was 65.6%. Seven concentrations of tannin and a control (EtOH‐treated seeds) were set up. Three treated or control beans were placed in a 5 cm height × 2 cm diameter vial with three females and three males, 3–4 days old. There were 39 replicate vials per treatment. The experiment lasted for 6 days with the vials being kept in complete darkness at 23°C. Vials of each concentration were placed on separate trays, but the position of trays in the controlled environment and the position of vials on trays was completely randomised for the experimental design. After the 6 days, the number of eggs/vial was counted. The model was fitted using the method of nonlinear least squares in Genstat. For details, see Suppl_3.

Tannin (MW 1701.23) had a particularly strong effect on *A. obtectus* egg‐laying (with small variation and the greatest *t*‐statistic observed, Table 2) and demonstrated a concentration‐dependent response from females (Figure 3). Oviposition response to surface treatment showed a highly significant (*p* < .001, *F*‐test) effect of tannin concentration, with a dose greater than 0.004 M required for there to be a major response and doses greater than 0.012 M being increasingly inhibitive to egg laying. Note that 0.004 M application equates to approximately 0.04 mg tannin applied onto one seed, which is approximately the same amount of tannin extractable from the testa of one seed (Reddy et al., 1985). Whole bean seed extracts are also oviposition stimulants (Monge, 1983), as is, in peculiar, magnesium sulphate (Szentesi, 1989). D‐catechin is an oviposition stimulant for *C. chinensis* L. (Ueno et al., 1990).

Szentesi (2021) postulates that the seed coat does not completely inhibit the detection of chemical cues from the seed cotyledon, thereby enabling egg‐laying *A. obtectus* females to assess substrate suitability for larval survival by integrating oviposition‐stimulating positive and larval development‐impeding negative chemical stimuli. Such infochemicals might include volatile organic compounds (Khelfane‐Goucem et al., 2014). A multiple‐choice experiment with a wide range of legume SPMs incorporated into artificial seeds shed light on the capacity of females to make oviposition choices if they can directly gain information about the chemical composition of the seed cotyledon. The differences in the influence on egg‐laying choices between SPMs become more pronounced at higher compound concentrations and their type of activity seems to be independent of their chemical class (Á. Szentesi, unpublished). Some of them, such as genistein, smilagenin, tropinone, nicotine H‐tartrate and digitonin, appear to be oviposition stimulants in a positive concentration‐dependent manner, whereas the most dramatic negative response with increasing concentration is seen for cinnamic acid (see Suppl_4). Coumarin and vanillin are oviposition repellents.

The importance of chemical cues in the oviposition decision‐making of *A. obtectus* is thought to be reflected in the preference order of host and acceptable non‐host seeds (categories by de Boer & Hanson, 1984). Females clearly rank dry legume seeds based on a hierarchy threshold model (Courtney et al., 1989), for which the rank‐order of hosts is invariable. Oviposition occurs when acceptability of a substrate exceeds the individual motivational threshold determined by genetic and physiological status, and the different acceptability levels create a rank order. Variation in egg‐laying is also modulated by factors such as egg load (Szentesi, 2021) and is modified by learning (Á. Szentesi, unpublished).

### 2.4 Entering the seed and larval development

Because L1 larvae of *A. obtectus* have legs and are thus mobile (an ancient character compared to more derived bruchid lineages, Pfaffenberger & Johnson, 1976), they can actively choose between seeds (Vuts, Woodcock, König, et al., 2018). This situation only occurs in granaries, where several legume species may be stored together, whereas the choices the larvae face in the field are restricted to differences in seed size and quality within a pod of a single host species. The seed testa of the primary host, *P. vulgaris*, presents an effective physical barrier, with more than a quarter of L1 larvae dying outside the beans; however, if the seed coat has pre‐drilled artificial entry holes, larval mortality is significantly lower (Szentesi, 2021). This is in part due to seed coat thickness (host and acceptable non‐host seeds have thinner testa; Szentesi, 2021), toughness (water content; Thiéry, 1984), but also chemical composition. Bean seed surface chemistry stimulates larval drilling into the seed by providing host recognition cues (Thiéry et al., 1994), but the seed coat material itself is spat out by chewing larvae because of the presence of toxic substances. This behaviour could be adaptive, which Stamopoulos (1988) links to the lignin content of the testa, but other compounds (e.g., phaseolin, vicilin, tannic acid, tannins) may also be responsible for seed coat indigestibility (see Suppl_5). Some of these compounds can reach high concentrations in legume seeds: tannins comprise 5% of the dry weight of *Vicia faba* seed testa (Boughdad et al., 1986), whereas phaseolin can reach as much as 16.7% dry weight in *P. lunatus* (Moraes et al., 2000). Seed coat toxins might partially be the reason why L1 larvae prefer to enter the seed via holes pre‐drilled by conspecifics (Labeyrie, 1960; Ohtsuka & Toquenaga, 2009).

Larval development to adulthood is ultimately determined by the chemical composition of the cotyledon and is affected by the presence of SPMs, although it is appreciated here that no clear distinction exists between so‐called primary and secondary plant metabolites (Erb & Kliebenstein, 2020). The taxonomic distribution of SPMs restricts the range of legume species in which *A. obtectus* can complete its life cycle, reflecting the breadth of the species' detoxifying capacity. Concerning the SPMs that occur in *Phaseolus* seeds (see Suppl_5), a series of experiments with artificial seeds incorporating a selection of synthetic compounds established that *A. obtectus* larvae are able to metabolise a relatively wide range of phenolic acid derivatives, phenolic glycosides, flavonoids and even some alkaloids, whereas many non‐protein amino acids appear to be toxic to them (Tables 3, 4, 5, 6; see Suppl_6 for the selected compounds and their sources; Á. Szentesi, unpublished). Alkaloids occur in 0.1%–0.4%, whereas phenolic glycosides occur in up to 30%, dry weight concentration in host and acceptable non‐host seeds of *A. obtectus*, and many of such host species have undergone domestication to reduce SPM concentrations and increase nutritional value and flavour (Szentesi, 2021). The consequence of this process may be reflected in the case of non‐protein amino acids, which can account for 1%–8% of seed dry weight (Bell & Tirimanna, 1965). Of them, for example, L‐canavanin occurs in certain *Vicia* species, which do not support *A. obtectus* development (Szentesi, 2021), but not in *V. faba*, which does. L1 larvae appear to cope better with some other non‐protein amino acids, for example, diaminopropionic acid or homoarginine, which characterise *Lathyrus* species (Bell, 1972) and in which *A. obtectus* can complete its life cycle (Szentesi, 2021). It is important to note, however, that SPMs toxicity may be affected by synergistic and/or antagonistic interactions among different compounds (Janzen et al., 1977), highlighting the limitation on conclusions to be drawn from tests with individual compounds (Whitaker et al., 2022). Recent approaches to build up *Phaseolus* resistance to *A. obtectus* focus on increasing seed APA (arcelin, phytohemagglutinin and α‐amylase inhibitor) protein content (Velten et al., 2008; Zaugg et al., 2013), which interferes with digestion in different ways (Sales et al., 2000).

**TABLE 3:** The highest concentrations of alkaloids at which adult emergence of *Acanthoscelides obtectus* was recorded from artificial beans incorporated with compounds occurring in seeds of leguminous plant species.

Alkaloids	Concentration (w/w%)	Emergence of adults (%)
Salsolidine	0.1	35.7
Tyramine	0.1	12.6
Control 1	0	10.7
Sparteine	0.01	1.2
Control 2	0	11.5

*Note*: No adult emergence occurred from artificial seeds having cytisine, lupinine, eserine, betonicine, gramine, crotaline, trigonelline and tryptamine (Á. Szentesi, unpublished). Into the powder of pulverised cotyledon of Valja bean variety, water‐soluble potato starch powder was mixed, max. 5%, which then was substituted with salsolidine or tyramine in 0.1% in order to keep the quantity of beans constant (known as the ‘wet’ method). The pulverising device was a Tekmar® A‐10 grinder (IKA, Staufen, Germany), cooled with cold water to avoid degradation of seed ingredients at 20,000 rpm. Only the cotyledon was pulverised, because based on literature data (Stamopoulos & Huignard, 1980) and our own experience, bean seed testa is toxic to L1. The artificial seeds (pilules) were formed with a pharmaceutical device and dried at 40°C for a day. Each of them was approximately 150 mg, supporting the development of one *A. obtectus* larva into the adult stage. One pilule was placed into a 60 mm × 10 mm glass vial with one fertile egg, which was closed with a cotton plug (*n* = 51/compound, corresponding with control 1). To test the effect of sparteine, 1 g tablets (13 mm diameter, 5 mm thickness) were prepared by mixing 99% cotyledon powder of Valja bean variety with 1% potato starch powder (control pilules) and substituting the latter with 0.0%, 0.01% or 1% SPM using a hydraulic press with 15 tons of pressure to achieve hardness similar to that of natural beans (known as the ‘dry’ method). Control tablets consisted only of cotyledon powder of Valja bean variety. Each tablet was cut into four sections, each sufficient for the development of one larva. The sections were placed into a 60 mm × 10 mm glass vial with one fertile egg, which was closed with a cotton plug (*n* = 15/compound, corresponding with control 2).

**TABLE 4:** The highest concentrations of non‐protein amino acids at which adult emergence of the seed beetle (*Acanthoscelides obtectus*) was recorded from artificial beans incorporated with compounds occurring in seeds of leguminous plant species.

Non‐protein amino acids	Concentration (w/w%)	Emergence of adults (%)
L‐Abrin (taxalbumin)	0.1	3.1
L‐canavanine	1.0	4.0
beta‐cyano‐L‐alanine	0.01	6.3
DL‐2,3‐diaminopropionic acid	0.01	40.6
L‐2,4‐diaminobutyric acid	0.1	3.1
L‐djenkolic acid	0.1	18.8
L‐3,4‐dihydroxyphenylalanine	0.01	6.5
D‐3,4‐dihydroxyphenylalanine	0.1	3.1
L‐homoarginine	0.1	9.4
L‐homoserine	0.1	9.4
N‐(p‐hydroxyphenyl)‐glycine	0.1	6.3
L‐5‐hydroxy‐tryptophan	0.01	10.0
DL‐α‐methyl‐glutamic acid	0.1	6.3
DL‐pipecolic acid	0.1	13.3
Control	0	38.5

*Note*: No adult emergence occurred from artificial seeds having L‐mimosine and β‐aminopropionitrile fumarate (Á. Szentesi, unpublished). Pilules were prepared by the ‘dry’ method (see Table 3; *n* = 32/compound).

**TABLE 5:** The highest concentrations of phenols and phenolic glycosides at which adult emergence of the seed beetle (*Acanthoscelides obtectus*) was recorded from artificial beans incorporated with compounds occurring in seeds of leguminous plant species (Á. Szentesi, unpublished).

Phenolic acid derivatives and phenolic glycosides	Concentration (w/w%)	Emergence of adults (%)
Coumarin	0.1	0
Gallic acid	1.0	41.0
Tannin	0.1	76.0
Condensed tannin	0.1	33.0
Umbelliferone	5.0	3.0
Vanillin	5.0	10.0
*p*‐Arbutin	5.0	33.0
Aesculin	5.0	33.0
Control	0	40.0

*Note*: Pilules were prepared by the wet method (see Table 3; *n* = 51/compound).

**TABLE 6:** The highest concentrations of flavonoids and flavonoid glycosides at which adult emergence of the seed beetle (*Acanthoscelides obtectus*) was recorded from artificial beans incorporated with compounds occurring in seeds of leguminous plant species (Kim et al., 2021; Á. Szentesi, unpublished).

Flavonoids and flavonoid glycosides	Concentration (w/w%)	Emergence of adults (%)
Morin	5.0	2.0
Naringin	5.0	47.0
Genisteine	0.1	7.0
Rutin	5.0	47.0
Rotenone	0.01	15.6
Control	0	43.6

*Note*: Pilules were prepared by the ‘wet’ method (see Table 3; *n* = 51/compound).

## 3 CHEMICAL ECOLOGY IN THE FIELD

According to Szentesi (1990), adult *A. obtectus* leave overwintering sites in Hungary in late May and feed on pollen and nectar of a range of plants until August when bean pods normally mature and oviposition begins. It is known from field trapping trials that adults are present already at the blooming stage of beans (Vuts et al., 2021). In a study by Jarry (1987), Poaceae pollen comprised 60% of the total pollen isolated from the digestive tract of members of a French *A. obtectus* population, whereas the percentages for Amaranthaceae and Apiaceae were 20% and 6%. This is surprising, because these bruchids are normally observed feeding on umbelliferous plants, thus Jarry (1987) suggests that the large proportion of ingested grass pollen is due to its high abundance among the vegetation frequented by the beetles, so that it is being eaten by them mostly by accident. Pollen has a high protein content plus sugar, starch, fat, and traces of vitamins and inorganic salts, while nectar primarily consists of a solution of sugars, especially glucose, fructose and sucrose (Wäckers et al., 2007). Laboratory feeding experiments with female *A. obtectus* have demonstrated that pollen consumption stimulates ovary production (Huignard & Leroi, 1981). Similarly, obligatory pre‐copulation feeding on pollen was reported in the bruchid *Bruchus pisorum* L. on *Pisum sativum* L. (Leguminosae; Pajni, 1981), as well as nectar feeding to obtain a readily available source of energy to sustain flight (Clement, 1992). The cues from flowers governing plant–bruchid interactions are, however, poorly understood. Zachariae (1958) lists nectar plants that all appear white or light yellow to the human eye, and traps coloured white or yellow were found to be the most attractive to *A. obtectus*. Other cues, such as odour, can also be pivotal for the locating of nectar plants by bruchid beetles.

### 3.1 Olfaction

*Bruchus rufimanus* is often found in flowers of *V. faba* L. (Leguminosae) and is attracted in the field to a synthetic mixture of the *V. faba* floral scent constituents (*R*)‐linalool, cinnamyl alcohol and cinnamaldehyde, as identified from headspace extracts (Bruce et al., 2011). Vuts, Woodcock, Caulfield, et al. (2018) studied the chemically guided relationships between *A. obtectus* and one of its nectar plants, *Daucus carota* L. (Apiaceae). Six EAG‐active flower headspace constituents (α‐pinene *S:R* 16:1, sabinene, myrcene, limonene *S:R* 1:3, terpinolene and [*S*]‐bornyl acetate) were isolated and identified, and their synthetic blend was found to induce behavioural preference in virgin females in laboratory olfactometer tests.

In contrast with the above‐described ecologically guided approach, screening of a broad range of ubiquitous floral compounds led to the discovery of the first effective field lure of *A. obtectus*. Because it visits several flowering plant species, Vuts et al. (2021) assumed that volatiles shared across the floral bouquet of the nectar plants favoured by *A. obtectus* are suitable candidates as generic attractants. Of the 27 compounds screened in EAG, five elicited sufficiently large antennal responses to be considered for further testing in olfactometer assays. Of these, only benzyl alcohol and methyl anthranilate were behaviourally preferred by the beetles, and a subsequent series of field trapping trials between early July and early September revealed benzyl alcohol to be an attractant of *A. obtectus*. Traps used in the field experiments in Vuts et al. (2021) caught only between 87 and 153 individuals in total, which may reflect usual population sizes in the study area (East Hungary) and also highlights the need for a wide‐range trapping campaign once the optimal trap design and lure composition are available. Although considered as a somewhat cruder approach than the traditional sequence of plant headspace analysis and identification of bioactive constituents (Bruce et al., 2011; Tewari et al., 2015), EAG and behavioural (laboratory and field) screening of a panel of candidate compounds has proven to be an effective way of discovering semiochemicals.

As the ecology and evolution of bruchids are ultimately linked with their larval host plants (Delobel & Delobel, 2006; Jermy & Szentesi, 2003; Kergoat et al., 2011), it is reasonable to suggest that studying the detection of host‐derived volatiles by egg‐laying females may yield powerful attractants to be deployed in pest detection and monitoring programmes. Ceballos et al. (2015) identified a range of volatiles from *P. sativum* leaves, flowers and pods, and demonstrated that headspace extracts evoked positive responses from *B. pisorum* in behavioural assays, but the compounds responsible for the bioactivity of the extracts are as yet unknown.

It is unknown how *A. obtectus* locates its host plant. Specimens overwintering in the environment of their host might search in a random manner, whereas those emerging from granaries may be able to detect volatile blends emitted by bean plants growing nearby. Labeyrie (1990) demonstrated this using large (several hectare) maize fields, within which randomly growing bean plants were successfully colonised by bruchids. Pouzat (1981) showed the EAG activity elicited by the vapour of bean pods, as well as by synthetic amyl acetate. Furthermore, dry bean seeds elicited positive behavioural responses from gravid females in olfactometer tests compared to blank air (mean time spent [min] ± SE, 3.6 ± 0.5 for seeds and 1.25 ± 0.3 for blank air; J. Vuts, unpublished), indicating the emanation of volatile kairomones from the seeds. Khelfane‐Goucem et al. (2014) correlated the behavioural responses of *A. obtectus* to volatile profiles of dry bean seed varieties and argued that the beetles differentiate between varieties using specific ratios of terpenoids, such as limonene and linalool. It is predictable, however, that husk volatiles at the stage when seeds are nearly mature within pods serve as potent kairomones for ovipositing females in the field and thus may offer potential for development of attractants. Fernandes and Nagendrappa (1979) report on the attractive properties of C_11_–C_24_ homologous fatty acids and their methyl esters, extractable from the pod surface of *Lablab purpureus* L., for one of its pests, *Adisura atkinsoni* Moore (Lepidoptera: Noctuidae).

Besides kairomones utilised as host‐derived attractants by insect herbivores, allomones make up a functional class of allelochemicals that elicit a negative response in the receiver relative to the emitter. The use of such repellent molecules, in the form of essential oils, has been explored in bruchid management. Papachristos and Stamopoulos (2002) found the vapour of mint, lavender and rosemary to be highly repellent to *A. obtectus*, as well as to reduce fecundity and larval emergence and survival. However, these complex volatile blends caused the evolution of resistance within eight generations (Papachristos & Stamopoulos, 2003). Essential oils and their volatile components (e.g., terpenoids) show repellent or attractive properties depending on dose. The impact of essential oil vapours on non‐target organisms and human health needs to be carefully evaluated to optimise dosage during the development of new fumigation agents (see Haddi et al., 2020 for overview).

### 3.2 Contact chemoreception

**FIGURE 4:**
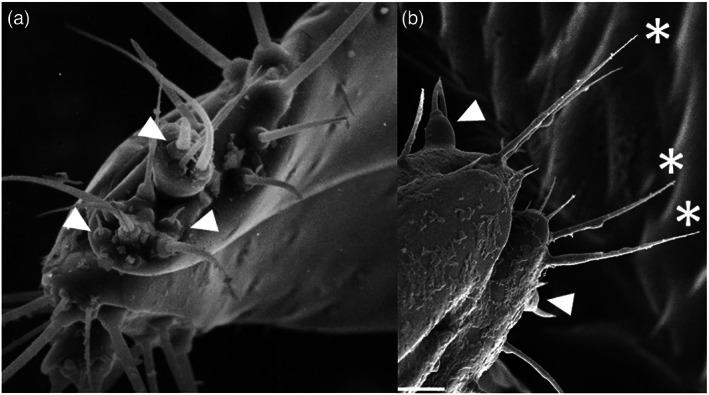
SEM image of the ovipositor tip of *Acanthoscelides obtectus*, showing putative chemoreceptors (arrows) and mechanoreceptors (asterisks). Source of Figure 4a: Szentesi (1976). Micrograph (b) was captured on a JEOL JSM6360LV Scanning Electron Microscope (Bioimaging Facility, Rothamsted Research). The white bar at the bottom of (b) represents 20 μm. For details, see Suppl_1.

Ovipositing insects assess the suitability of a substrate for larval development by using a range of physical and chemical cues. *A. obtectus* lays its eggs into nearly ripe bean pods after chewing a hole in the pod wall and inserting the ovipositor into the pod cavity, which assesses the internal environment. Females lay more eggs on large seeds than on small ones when given the choice, and it is assumed that in the field, pod curvature and size physically inform them on the resource size, that is the amount of seeds within pods (Szentesi, 2003), as is the case for other bruchids (Avidov et al., 1965; Messina, 1984). Such physical properties, together with pod moisture content, may be perceived by mechanosensory hairs and hygroreceptors on the tip of the ovipositor (Figure 4). Chemical cues provided by the seed coat are also important for host assessment as evidenced by Szentesi (1976) in ablation experiments. These showed that the maxillary palpi are the most important head appendages for chemical sensation of oviposition sites, whereas the antennae have a role primarily in shape recognition. Interestingly, a certain level of autonomy of the ovipositor in oviposition site selection was also revealed and putative chemosensilla on its tip identified (Figure 4). The author emphasises that further histological and physiological investigations are necessary to clarify the exact chemosensory mechanisms involved with the ovipositor (see Li et al., 2020; Yadav & Borges, 2017).

A series of experiments carried out with artificial bean seeds demonstrated a hierarchy of chemical factors for eliciting oviposition in *A. obtectus* (Á. Szentesi, unpublished). The most preferred egg‐laying substrate was bean cotyledon powder plus potato starch (control) spiked with powdered pod wall (Table 7), which underlines the importance of husk chemical cues in host recognition and acceptance. The role of seed coat chemicals might be restricted to oviposition substrate recognition and arrestment.

**TABLE 7:** Assessment of the importance of bean seed and pod chemistry in *Acanthoscelides obtectus* oviposition substrate recognition and acceptance.

Treatments	No. eggs laid/female (*n* = 13)	SD/SE	*F*‐ and *p*‐values, LSD (5%)
Cotyledon powder +20% soluble potato starch	7.64	8.40/2.33	27.7662, *p* < .001, LSD = 6.37 (36 df)
Artificial bean made from a whole powdered seed	13.95	6.80/1.90	
Cotyledon powder +20% bean pod powder	30.31	8.70/2.40	

*Note*: Artificial egg‐laying substrates (‘seeds’ or pilules) were made as follows: into the powder of pulverised (Tekmar® A‐10 grinder, Germany, cooled with cold water to avoid degradation of seed ingredients, at speed 20,000 rpm) bean cotyledon (var. Valja), water‐soluble potato starch powder was mixed to give a control preparation of bean cotyledon at 80% and water‐soluble potato starch at 20%. Another preparation was made with 20% starch substituted with bean pod powder. Having wetted with distilled water, the resulting pastry was rolled out, cut into pieces and finally formed by hand into 6 mm diameter balls. These were dried at 40°C for 1 day. In general, each pilule was approximately 150 mg. Three pilules with the same treatment were placed in a vial (5 cm height × 2 cm diameter), then three female and three male beetles were inserted. The vials were then left in darkness for 6 days at 23°C before counting the number of eggs laid (*n* = 13/treatment). A completely randomised design was used. Analysis of variance (ANOVA) was applied to test the significance (*F*‐test) of differences between the treatments (mean eggs laid). No transformation of the data was required, the residuals from the analysis showing that the data on the raw scale conformed to the assumptions of analysis (Levene and Brown‐Forsythe tests). Analysis used Statistica 8.0 (StatSoft, Inc., 2007). Note, least significant difference (LSD) at 0.1% = 11.27.

*Acanthoscelides obtectus* differs from many other bruchids in that it oviposits into nearly ripe pods and the mobile larvae choose suitable seeds to enter. In contrast, members of the so‐called ‘greed‐pod’ sub‐guild of pre‐dispersal seed predators (Szentesi & Jermy, 1995), such as *B. pisorum* L., glue their eggs onto the outer surface of the carpel of young green pods. As a result, tumour‐like growths of undifferentiated cells (neoplasms) develop beneath the egg in lines of *P. sativum* that exhibit the neoplastic pod phenotype, impeding entry of the legless larvae into the pod (Doss et al., 2000). The active substances that stimulate cell division at the sites of egg attachment are called bruchins, long‐chain α,ω‐diols esterified at one or both oxygens with 3‐hydroxypropanoic acid. Bruchins have so far been identified from *B. pisorum* (Doss et al., 2000) and *Callosobruchus maculatus* F. (Oliver et al., 2000). Clearly, the ability to perceive and react to these compounds bears some adaptive value for the individual plant, as it reduces seed damage, but the character that confers resistance (neoplasm formation) is not herbivore‐specific and is also observed in another legume genus (Doss et al., 2000). It is unknown if *A. obtectus* also produces bruchins with molecular structures similar to those described from *B. pisorum*. The production of bruchins might be an ancient character of the Bruchidae with yet unknown functions (e.g., glueing eggs onto host pod/seed, host‐marking pheromone), or with none. They are seemingly non‐adaptive for the beetles because of increased egg and larval mortality, but this trait may not be under selection pressure because of the lack of population‐level negative impact.

## 4 PREFERENCE–PERFORMANCE INTERACTIONS AND HOST SPECIALISATION

*Acanthoscelides obtectus* is an oligophagous host specialist that, like other insect herbivores, is assumed to maximise its fitness, measured as the number and quality of subsequent generations, by laying eggs on substrates that are most suitable for larval development (preference–performance hypothesis; Thompson, 1988). In general, as a result of host specialisation, fitness of specialist herbivores on acceptable non‐hosts is lower than on host plants, which is explained on the genetic level by trade‐offs among genes (antagonistic pleiotropy; Scheirs et al., 2005). This state is maintained by stabilising selection until, via autonomous changes (mutations) of the genome, an extreme phenotype appears that accepts the new host behaviourally and lays eggs on it, and then it evolves to metabolise SPMs and complete its development (Caillaud & Via, 2000; Jermy, 1984). The behavioural level of host shifts is linked to phenotypic plasticity, whereas the physiological processes require genetic changes in detoxification capabilities (West‐Eberhard, 2005). Due to their labile nature, behavioural characters change first, followed by those affecting physiology (e.g., Ikonen et al., 2003; Wasserman & Futuyma, 1981; West‐Eberhard, 2005). The host range of bruchids is determined predominantly by female egg‐laying behaviour that ensures the availability of high‐quality hosts for larval development (Siemens et al., 1991), although larvae can survive in more host plant species than the mother's preference suggests (Janzen, 1981). *A. obtectus* females have narrow host acceptance, which reflects a genetically fixed host rank order and allows fitness maximisation only on the most suitable larval hosts (Jermy & Szentesi, 1978). However, other legume species that support larval development (e.g., Tucić et al., 1997) indicate constant attempts to broaden host range. Compared to most bruchids, *A. obtectus* L1 larvae are mobile and can choose between seeds in granaries, whereas other species are unable to leave the plant their mother chose for their development. The female's oviposition onto suboptimal substrates during host shift may reflect the limited neuronal capabilities of insects (Bernays, 2001), forcing her offspring to either die or adapt. Ultimate adaptation on a new host occurs when there is positive genetic correlation (non‐random association) between alleles of genes on different loci that determine maternal preference–larval performance relationships, that is there must be genetic covariance between preference and performance to adapt to a new plant species (linkage disequilibrium; Fry, 1996). This is a likely scenario for specialist herbivores, the detoxification capacity of which is larger than that of generalists, and hence a host shift may require complex genetic changes to generate host races. Generalists, on the other hand, may undergo changes in host preference only at the phenotype level (=behaviour), which does not necessitate genetic linkages between preference–performance processes (Forister & Jenkins, 2017).

**FIGURE 5:**
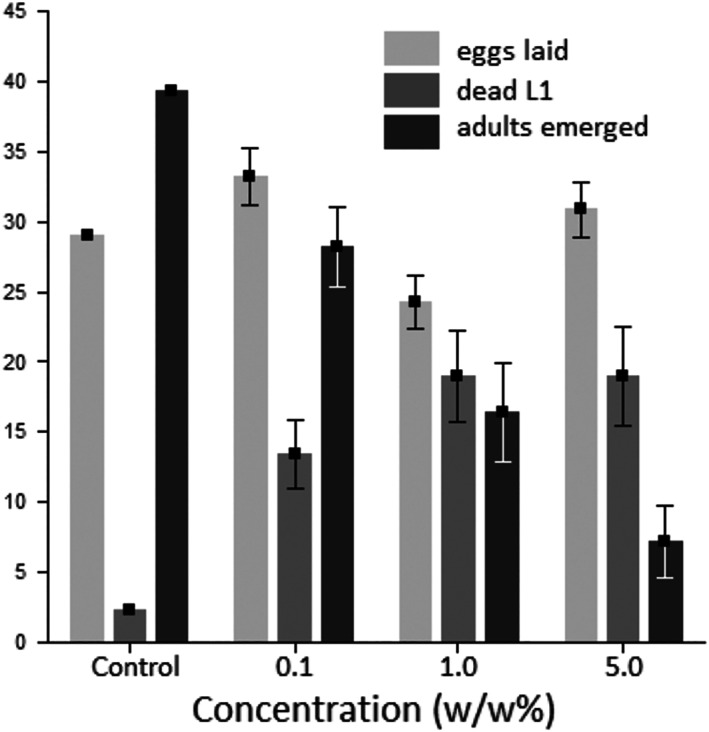
Mean (±SE) number of eggs/female laid on artificial seeds (light grey columns), the number of first instar larvae dead outside artificial seeds (dark grey columns) and the number of adult seed beetles (*A. obtectus*) emerged from artificial seeds (black columns) incorporated with various SPMs at 0.1, 1.0 and 5.0 w/w% concentrations. Responses are averages of 32 compounds at each concentration. (See Suppl_6 for compound names). No. of eggs: *F*_3,93_ = 3.6629, *p* = .0152, LSD_5%_ = 5.5. No. of L1 mort: *F*_3,93_ = 0.9629, *p* = .4137, LSD_5%_ = 8.75. No. of adults: *F*_3,93_ = 8.7346, *p* = .00004, LSD_5%_ = 8.45. Analysis used Statistica 8.0 (StatSoft, Inc., 2007).

**FIGURE 6:**
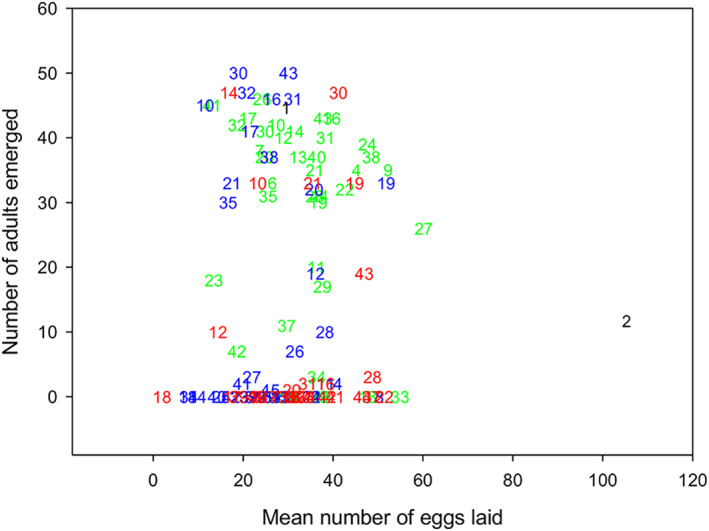
Number of *Acanthoscelides obtectus* adults emerged versus mean number of eggs laid. Average number of eggs laid by three females over seven replicate vials per treatment (SPMs at three concentrations [0.1%, 1% and 5%] and controls) was correlated with averages of subsequent adult emergence for the same treatments. Fifty‐one replicate vials, each containing a single egg, were set up per treatment (see information provided with Table 3). Development was studied over time (days). Numbers on the graph denote compounds applied to artificial seeds: 1 average control, 2 average bean control, 3 glass bead control, 4 L‐canavanine, 5 hordenine sulphate, 6 condensed tannin, 7 tannin, 8 picrotoxin, 9 quinidine sulphate, 10 syringic acid, 11 smilagenine, 12 vanillin, 13 theophilline, 14 naringin, 15 eserine sulphate, 16 morin, 17 gallic acid, 18 coumarin, 19 p‐arbutin, 20 homoprotocatechuic acid, 21 aesculin, 22 caffeine, 23 digitonin, 24 veronal Na, 25 reserpine, 26 hecogenine, 27 cinnamic acid, 28 umbelliferone, 29 tomatine, 30 rutin, 31 salicin, 32 isatin, 33 brucine, 34 strychnine, 35 quinine HCl, 36 ergotamine tartrate, 37 codeine, 38 cis‐aconitic acid, 39 nicotine hydrogen tartrate, 40 sodium oxalate, 41 tropinone citrate, 42 atropine, 43 genistein, 44 solasodine, 45 vincamine. Differently coloured numbers refer to 0.0 (black), 0.1 (green), 1.0 (blue) and 5.0 (red) w/w% concentrations. For details, raw data and further life parameter comparisons (L1 mortality, adult dry weight, development time), see Suppl_7 and Suppl_8.

Szentesi (2021) demonstrated that host range expansion of *A. obtectus* is unlikely, despite positive correlation between maternal preference and larval performance for several acceptable non‐host seeds in lab tests, because the recognition of a diverse set of seed pod‐related compounds would be necessary to induce egg‐laying in nature. A series of subsequent experiments using 32 synthetic compounds of a wide set of chemical classes (see Suppl_6 for compound names) that occur in seeds of host and acceptable non‐host plants of *A. obtectus* aimed at characterising how individual chemicals from legumes influence preference–performance relationships. When the effects of all compounds were considered together, females preferred to lay eggs equally across all concentrations, whereas larval survival decreased as concentrations increased (Á. Szentesi, unpublished; Figure 5). Despite the limitations posed by testing artificial seeds incorporated with only single compounds, the experiments revealed that the specialist herbivore *A. obtectus* can metabolise a large number of chemicals, although the discrepancy between oviposition and development patterns is intriguing at this level of analysis. The negative correlation coefficient in Table 8 reveals a so‐called preferential trade‐off at 0.1% compound concentration, indicating that L1 larvae hatching from eggs managed to enter the ‘seeds’ in most cases, but their development was negatively affected depending on individual chemicals. For example, brucine (compound number 33), an alkaloid, promoted strong egg‐laying at 0.1% concentration but hampered larval development, whereas coumarin (number 18) both strongly deterred oviposition and inhibited larval development (Figure 6). The varied responses do not highlight chemical groups associated with consistent patterns of development. Furthermore, the lack of correlation between maternal preference and larval performance at 0.1%, 1% and 5% concentrations was simply the result of L1 larvae dying outside the seeds before entering them, as they were not able to excrete, catabolise or sequester the incorporated chemicals at these doses (Table 8). Further experiments with groups of compounds would shed more light on the role of SPMs in *A. obtectus* preference–performance relationships. This would also help the explanation of how plant SPMs influence life strategies along the generalist–specialist continuum.

**TABLE 8:** Pearson correlation coefficients (*r*) between adult preference (measured as mean number of eggs laid) and larval performance (number of emerged adults).

Interaction	Correlation coefficient, *r*	Means (eggs/adults)	*n*
Mean number of eggs laid versus number of adults emerged (at 0.1%, 1.0% and 5.0% concentrations)	0.0375	29.8/17.2	117
Mean number of eggs laid versus number of adults emerged at 0.1% concentration	−0.1129	33.0/26.4	43
Mean number of eggs laid versus number of adults emerged at 1.0% concentration	0.0481	25.1/14.8	40
Mean number of eggs laid versus number of adults emerged at 5.0% concentration	0.0149	31.3/8.4	34

*Note*: Artificial seeds were incorporated with various SPMs at 0.1, 1.0 and 5.0 w/w% concentrations. Responses are averages of 32 compounds at each concentration. (See Suppl_6 for compound names). *F*‐test of the correlation for all three concentrations: *F*_1,115_ = 0.162, *p* = .6881; 0.1%, *F*_1,41_ = 0.5292, *p* = .4711; 1.0%, *F*_1,38_ = 0.088, *p* = .7683; 5%, *F*_1,32_ = 0.0071, *p* = .9332. Analysis used Statistica 8.0 (StatSoft, Inc., 2007).

Around 70% of herbivorous insects, including all of the Bruchinae, is host‐specialist, developing on one plant species (monophagous) or one plant family (oligophagous; Forister et al., 2015). Plant–insect relationships are predominantly determined by the distribution of SPMs (i.e., the ‘plant phytochemical profile’) and the insect's behavioural responses to them (Jermy & Szentesi, 2021); bruchid host specialisation is ultimately determined by plant traits at tribe level (Jermy & Szentesi, 2003; Kergoat et al., 2005), including characteristic chemical groups, the distribution of which, however, is less consistent in the Leguminosae (Wink & Mohamed, 2003). Behavioural outputs are dependent on sensory information elicited by a combination of positive and negative (inhibiting) stimuli, the latter exerting a bigger impact on stimulus integration outcomes and thus responses to encountered plants (Dethier, 1982; Schoonhoven et al., 1997). Specialist herbivores are deterred by many SPMs but metabolise the toxins in their own host plants very efficiently and can even use them for host recognition. They have evolved by adapting to those SPMs that have appeared in plant phylogenetic lineages and the detoxification of which required key innovations on behalf of the herbivore. For example, the Pierinae (Lepidoptera: Pieridae) are thought to have radiated on crucifers after acquiring glucosinolate‐detoxifying mechanisms (Wheat et al., 2007), and Rosenthal (1983) proposed the bruchid *Caryedes brasiliensis* Thunberg to have developed biochemical mechanisms to adapt to the L‐canavanin content of its host seed (*Dioclea megacarpa* Rolfe), including sequestration and detoxification processes. Generalists, on the other hand, utilise the inducible capacity of their microsomal oxidase system to handle the plethora of encountered SPMs (Castells & Berenbaum, 2008). Inhibitory stimuli have been demonstrated to play a dominant role in *A. obtectus* oviposition, asymmetrically influencing egg‐laying choices (Jermy & Szentesi, 1978).

Multiple theories attempt to explain the mechanisms that create the patterns of host use along the generalist–specialist continuum. These differ from each other by the amount of emphasis placed on the importance of (i) adaptation, (ii) phylogenetic conservatism, (iii) ecological speciation, (iv) random events, or (v) autonomous mutations and drift. Although generally assumed, specialisation of herbivorous insects does not necessarily need to be adaptive, and host specificity can be created by mutations and genetic drift without trade‐offs (specialisation by drift; Gompert et al., 2015; Hardy et al., 2016; Jermy & Szentesi, 2021). It will be a task of future studies to shed light on the likely specialisation mechanisms in the oligophagous *A. obtectus*.

## 5 CONCLUSIONS

**FIGURE 7:**
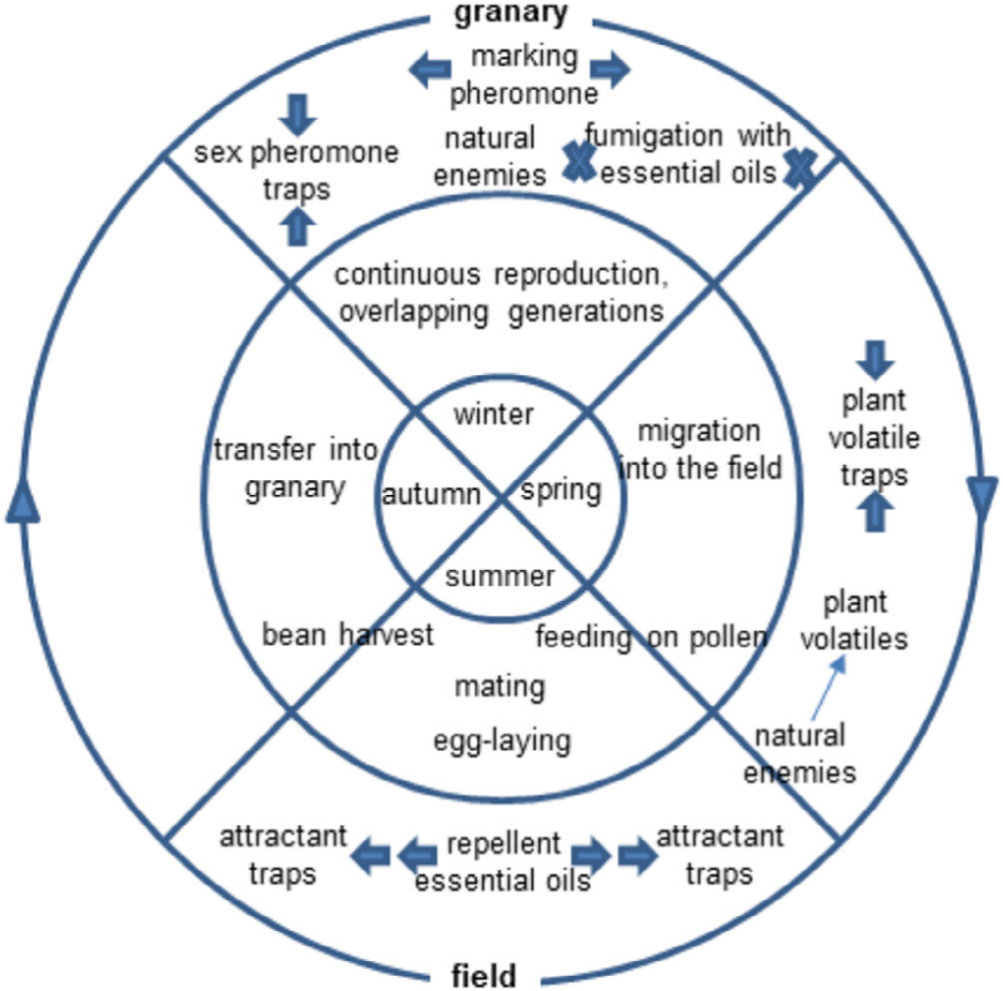
Scheme of semiochemical‐based intervention opportunities for *Acanthoscelides obtectus* management throughout the year in temperate Europe. Attractants cover both the sex pheromone and plant volatiles. The direction of arrows indicates attractive (towards label) or repellent (away from label) effects. Crosses next to essential oils symbolise lethal effects.

All the research that has uncovered the semiochemicals which govern the intra‐ and interspecific ecological interactions of *A. obtectus* can potentially lead to environmentally benign pest management strategies as part of integrated pest management (IPM) programmes. Semiochemicals are usually required at doses low enough not to be toxic (Witzgall et al., 2010) and can be used for detection, monitoring or mass‐trapping of target pests. A scenario that combines repellents (allomones) and attractants (pheromones or kairomones) is called a push‐pull system, and this has already been used successfully against *Alphitobius diaperinus* Panzer (Coleoptera: Tenebrionidae; Hassemer et al., 2019) for example. Such a system could also be used against *A. obtectus*; Figure 7 sums up the suggested options. Although the *A. obtectus* pheromone constituents are difficult to synthesise to make available for lures, the discovery of analogues that are more accessible could facilitate trap development, such as in the bruchid *Caryedon serratus* Olivier, which is attracted to hexadecanoic acid ester derivatives both in lab bioassays and trapping trials (Tewari et al., 2015). Interestingly, the active structures are similar to methyl (*E*,*R*)‐2,4,5‐tetradecatrienoate, the most abundant component of the *A. obtectus* sex pheromone. Current developments on floral volatile‐derived attractants could lead to compounds that synergise the activity of future pheromone lures or serve as bisexual lures. Ideally, in an IPM context, semiochemical‐based methods are combined with the breeding of bruchid‐resistant bean cultivars, appropriate storage facilities and, where absolutely necessary, insecticide applications. The recruitment of parasitoids from the local pool of natural enemies should also be considered particularly at the field scale, where they use host semiochemicals (Fatouros et al., 2005) or host‐induced plant volatiles (Felton & Tumlinson, 2008) as foraging cues.

Natural enemies of pests can reduce their populations below an economic threshold level, especially in isolated environments such as greenhouses (Pijnakker et al., 2020), and the use of parasitic wasps against stored‐product pests in granaries holds similar promises (Hervet & Morrison III, 2021). The biocontrol efficiency of a number of predators and hymenopteran parasitoids has been assessed. *Xylocoris flavipes* Reuter (Hemiptera: Anthocoridae) provided strong suppression of *A. obtectus* populations by feeding on eggs and early larval instars (Sing & Arbogast, 2008). *Dinarmus basalis* Rondani (Hymenoptera: Pteromalidae) was found to be a good candidate for the in‐store management of *A. obtectus* in combination with other approaches, such as enhanced seed resistance (Schmale et al., 2005; Velten et al., 2008). Furthermore, the pteromalid *Anisopteromalus calandrae* Howard combined with *Blattisocius tarsalis* Berlese (Acari: Ascidae) mites caused an 81% bruchid reduction in emergence (Iturralde‐García et al., 2020). Establishing the semiochemistry behind parasitoid host location (Mbata et al., 2004) can improve control efficiency and become a valuable tool for storage pest IPM (Trematerra, 2012). Of the parasitoids of *A. obtectus* in the Neotropics and Europe (Cox, 2007; de Luca, 1965; Schmale et al., 2002), *D. basalis* has been shown to use the oviposition‐marking pheromone of one of its hosts, *C. chinensis*, as a kairomone to locate infested seeds (Kumazaki et al., 2000); however, studies on *D. basalis* responses to *A. obtectus*‐related semiochemicals are lacking. A promising avenue might be to establish if volatile cues from infested seeds guide wasp host‐finding, which could lead to development of powerful lures to recruit natural enemies for mitigating pest pressure. Such recruitment may be limited to natural parasitoid populations and require artificial release of biocontrol agents, because *A. obtectus* shows low parasitisation in Europe perhaps due to its isolated life cycle under storage conditions (Á. Szentesi, pers. commun.). Other factors can also limit the negative impact of natural enemies on bruchids: the invasive *Acanthoscelides pallidipennis* Motschulsky sequesters rotenone from its host *Amorpha fruticosa* L. in Hungary, which makes it less palatable for consumers (Szentesi, 1999) and perhaps also reduces the success rate of its parasitoids.

Research has so far been concerned with specific aspects of the complex life cycle of *A. obtectus*. It is now time to combine the many valuable results into an IPM strategy. Besides the semiochemical‐based approaches (Figure 7), other control measures timed to the appropriate phase of its life cycle should be considered. Such measures need to include physical, chemical and biological interventions in a harmonious way. For instance, spreading of local infestation from small batches of seeds can be prohibited by post‐harvest deep‐cooling of seeds. Further management steps should rely upon the application of semiochemicals in the field, providing information on the presence, distribution, population size, dispersal, and so forth. of *A. obtectus*. We hope knowledge about the chemical ecology of *A. obtectus* collated in this paper will give a useful starting point for the development of IPM tools, which in turn can be adapted for other pest bruchids. This may prove especially useful in the light of the predicted prominence of legumes in future human diet.

## Supporting information


**Data S1.** Supporting information.

**Data S2.** Supporting information.

**Data S3.** Supporting information.

**Data S4.** Supporting information.

**Data S5.** Supporting information.

**Data S6.** Supporting information.

**Data S7.** Supporting information.

**Data S8.** Supporting information.
