# Supplementary material for: A semiochemical view of the ecology of the seed beetle Acanthoscelides obtectus Say (Coleoptera: Chrysomelidae, Bruchinae)
Source: Ann Appl Biol. 2023 Sep 4;184(1):19–36. doi: 10.1111/aab.12862 (PMC10953445; doi:10.1111/aab.12862)
Supplement: Supplementary file 1 — Data S1. Supporting information. [file AAB-184-19-s007.docx]

A semiochemical view of the ecology of the seed beetle *Acanthoscelides obtectus* Say (Coleoptera: Chrysomelidae, Bruchinae)

József Vuts, Stephen J Powers, Eudri Venter, Árpád Szentesi

**Correlations between egg laying and development characteristics**

**Data and Methods**

Average number of eggs laid by three females over seven replicate vials per treatment (SPS chemicals at three concentrations (0.1, 1 and 5%) and controls) from the no choice test experiment was correlated with averages of variables per treatment from a separate experiment to consider development of larvae and subsequent adult emergence for the same treatments. In this separate experiment, 51 replicate vials, each containing a single egg, were set up per treatment. Development was studied over time (days). The variables of interest measured were:

% L1 larval mortality outside of pilules

Adult emergence (%, out of 51)

Dry weight of adults (mg)

Number of adults emerged (out of 51)

Average development time (days)

Minimum development time (days)

Average development time of females (days)

Average development time of males (days)

Pearson’s correlation (invoking the F-test) was used to assess the strength of the relationships. For this exercise, to enable distributional assumptions, data were transformed to the log scale, or to the logit scale for percentages.

**Results**

There were no statistically significant (p < 0.05, F-test) correlations, when looking at all the data, or at data for each concentration separately. This result supports the situation that although the chemicals collectively represent a range, from inhibitors to enhancers, the situation is more complex when considering numbers of eggs laid and subsequent development together. Hence, plotting the development variables versus the average number of eggs laid reveals chemicals of interest, which may be picked out in relation to other chemicals that would appear to perform in a similar way as regards overall fecundity. The key for the numbers representing the controls and chemicals on the graphs which follow is:

**# Name**

1 Average control

2 Average bean control

3 Glass bead control

4 L-Canavanine

5 Hordenine sulphate

6 Condensed tannin

7 Tannin

8 Picritoxin

9 Quinidine sulphate

10 Syringic acid

11 Smilagenine

12 Vanillin

13 Theophilline

14 Naringin

15 Eserine sulphate

16 Morin

17 Gallic acid

18 Coumarine

19 p-Arbutin

20 Homoprotocatehuic acid

21 Aesculin

22 Coffeine

23 Digitonine

24 Veronal-Na

25 Reserpine

26 Hecogenine

27 Cinnamic acid

28 Umbelliferon

29 Tomatine

30 Rutin

31 Salicine

32 Isatin

33 Brucine

34 Strychnine

35 Quinine HCl

36 Ergotamine tartrate

37 Codeine

38 cis-Aconitic acid

39 Nicotine hydrogen tartrate

40 Sodium oxalate

41 Tropinone citrate

42 Atropine

43 Genisteine

44 Solasodine

45 Vincamine

**The control treatments are plotted in black, 0.1% concentration in green, 1% in blue and 5% in red.**

The four corners of this plot can be investigated for interesting chemicals.

Top left: bad for eggs, bad for larval survival;

Top right: good for eggs, bad for larval survival;

Bottom left: bad for eggs, good for larval survival;

Bottom right: good for eggs, good for larval survival.

Chemical 18 (Coumarine) is clearly the most lethal to egg-laying and larvae when at 1% and 5%, although at 0.1% it actually promoted a large number of eggs (average 47.1), albeit with 41.2% larval mortality. Chemicals 37 (Codeine) and 22 (Coffeine) at 5%, 8 (Picritoxin) at 1% and 33 (Brucine) at 0.1% promote large numbers of eggs to be laid but successful larval development from them is minimal. Chemicals 10 (Syringic acid) and 40 (Sodium oxalate) at 1% and 41 (Tropinone citrate) at 0.1% do not promote eggs but are good for larval survival. Chemical 27 (Cinnamic acid) at 0.1% promotes the greatest number of eggs combined with relatively low larval mortality.

The four corners of this plot can be investigated for interesting chemicals.

Top left: bad for eggs, good for body weight;

Top right: good for eggs, good for body weight;

Bottom left: bad for eggs, bad for body weight;

Bottom right: good for eggs, bad for body weight.

Chemicals 35 (Quinine HCl) and 41 (Tropinone citrate) at 1% and 10 at 5% appear to promote relatively low numbers of eggs but high dry weight of adults. Chemical 13 (Theophilline) at 0.1% is also of interest, with high dry weight albeit with a medium number of eggs. Chemicals 9 (Quinidine sulphate) and 27 (Cinnamic acid) at 0.1% are best for combined high eggs and relatively high dry weight of adults. Chemical 18 (Coumarine) at 5% both strongly deterred oviposition (on average only 1.4286 eggs laid, n = 7) and inhibited larval development and subsequent reduced dry weight of adults. Chemical 33 (Brucine) at 0.1% promotes eggs but not body weight.

The four corners of this plot can be investigated for interesting chemicals.

Top left: bad for eggs, good for adult emergence;

Top right: good for eggs, good for adult emergence;

Bottom left: bad for eggs, bad for adult emergence;

Bottom right: good for eggs, bad for adult emergence.

Chemicals 10 (Syringic acid), 30 (Rutin) and 32 (Isatin) at 1%, 41 (Tropinone citrate) at 0.1%, and 14 (Naringin) at 5% provided low numbers of eggs but successful emergence of adults. Chemical 43 (Genisteine) also provided good adult emergence, but with slightly greater eggs. Chemical 30 (Rutin) at 5% gave larger numbers of eggs and adults. Chemical 18 (Coumarine) at 5% was detrimental for both eggs and adults. Chemical 33 (Brucine) at 0.1% promotes eggs but not adult emergence. The bean control (2) gave the largest number of eggs overall but adult emergence was relatively low.

Raw data used for plot:

| Chemical | # | Concentration (w/w%) | | | | | |
| --- | --- | --- | --- | --- | --- | --- | --- |
|  |  | 0 (controls) or 0.1 | | 1.0 | | 5.0 | |
|  |  | Mean Eggs laid | Adults emerged | Mean Eggs laid | Adults emerged | Mean Eggs laid | Adults emerged |
| Average Control | 1 | 29.0000 | 44.6667 | - | - | - | - |
| Average Bean | 2 | 104.762 | 11.6700 | - | - | - | - |
| Glass bead | 3 | 17.7143 | * | - | - | - | - |
| L-Canavanine | 4 | 44.7143 | 35 | 40.5714 | 2 | 33.7143 | 0 |
| Hordenine sulphate | 5 | 28.7143 | 0 | 20.8571 | 0 | 22.5714 | 0 |
| Condensed tannin | 6 | 26.0000 | 33 | 28.0000 | 0 | * | * |
| Tannin | 7 | 23.1429 | 38 | 22.0000 | 0 | 26.7143 | 0 |
| Picritoxin | 8 | 30.5714 | 0 | 49.7143 | 0 | 31.4286 | 0 |
| Quinidine sulphate | 9 | 51.7143 | 35 | 23.4286 | 0 | 29.7143 | 0 |
| Syringic acid | 10 | 26.8571 | 42 | 11.0000 | 45 | 22.7143 | 33 |
| Smilagenine | 11 | 35.7143 | 20 | 37.8571 | 2 | * | * |
| Vanillin | 12 | 28.4286 | 40 | 35.5714 | 19 | 13.8571 | 10 |
| Theophilline | 13 | 31.7143 | 37 | 27.8571 | 0 | 30.1429 | 0 |
| Naringin | 14 | 31.0000 | 41 | 9.2857 | 0 | 16.3333 | 47 |
| Eserine sulphate | 15 | 24.0000 | 0 | * | * | * | * |
| Morin | 16 | 39.1429 | 43 | 25.8571 | 46 | 37.8571 | 2 |
| Gallic acid | 17 | 20.5714 | 43 | 21.0000 | 41 | 17.0000 | 0 |
| Coumarine | 18 | 47.1429 | 0 | 7.14286 | 0 | 1.42857 | 0 |
| p-Arbutin | 19 | 36.1429 | 30 | 51.2857 | 33 | 44.2857 | 33 |
| Homoprotocatehuic acid | 20 | 24.2857 | 37 | 35.2857 | 32 | 30.2857 | 1 |
| Aesculin | 21 | 35.4286 | 35 | 17.0000 | 33 | 35.0000 | 33 |
| Coffeine | 22 | 42.1429 | 32 | 34.7143 | 0 | 50.8571 | 0 |
| Digitonine | 23 | 13.0000 | 18 | 18.7143 | 0 | 33.4286 | 0 |
| Veronal-Na | 24 | 47.1429 | 39 | 34.7143 | 0 | * | * |
| Reserpine | 25 | 28.2857 | 0 | 14.5714 | 0 | * | * |
| Hecogenine | 26 | 23.7143 | 46 | 31.0000 | 7 | * | * |
| Cinnamic acid | 27 | 59.7143 | 26 | 21.4286 | 3 | 25.0000 | 0 |
| Umbelliferon | 28 | 35.4286 | 31 | 37.7143 | 10 | 48.2857 | 3 |
| Tomatine | 29 | 37.1429 | 17 | 25.0000 | 0 | * | * |
| Rutin | 30 | 24.4286 | 41 | 18.4286 | 50 | 40.7143 | 47 |
| Salicine | 31 | 37.8571 | 40 | 30.5714 | 46 | 33.8571 | 2 |
| Isatin | 32 | 18.1429 | 42 | 20.2857 | 47 | 32.0000 | 0 |
| Brucine | 33 | 54.4286 | 0 | 25.8571 | 0 | 31.4286 | 0 |
| Strychnine | 34 | 35.8571 | 3 | 7.42857 | 0 | 33.4286 | 0 |
| Quinine HCl | 35 | 25.0000 | 31 | 16.1429 | 30 | 29.2857 | 0 |
| Ergotamine tartrate | 36 | 29.7143 | 0 | * | * | * | * |
| Codeine | 37 | 29.1429 | 11 | 22.7143 | 0 | 48.1429 | 0 |
| cis-Aconitic acid | 38 | 48.0000 | 37 | 25.2857 | 37 | 22.8571 | 0 |
| Nicotine hydrogen tartrate | 39 | 36.4286 | 0 | 20.7143 | 0 | 18.4286 | 0 |
| Sodium oxalate | 40 | 35.8571 | 37 | 13.7143 | 0 | 22.5714 | 0 |
| Tropinone citrate | 41 | 12.5714 | 45 | 19.2857 | 2 | 40.0000 | 0 |
| Atropine | 42 | 18.1429 | 7 | 16.8571 | 0 | 38.1429 | 0 |
| Genisteine | 43 | 37.2857 | 43 | 29.5714 | 50 | 46.4286 | 19 |
| Solasodine | 44 | 36.4286 | 31 | * | * | 46.0000 | 0 |
| Vincamine | 45 | 37.1429 | 0 | 25.5714 | 1 | 33.7143 | 0 |

The four corners of this plot can be investigated for interesting chemicals.

Top left: bad for eggs, bad for development;

Top right: good for eggs, bad for development;

Bottom left: bad for eggs, good for development;

Bottom right: good for eggs, good for development.

Chemicals 12 (Vanillin) at 5% and 41 (Tropinone citrate) at 1% gave long average development time and small numbers of eggs. Chemicals 34 (Strychnine) at 0.1% and 4 (L-Canavanine) at 1% along with chemicals 28 (Umbelliferon) and 31 (Salicine) at 5% provided the longest average development time with relatively large numbers of eggs. Chemicals 10 (Syringic acid) and 30 (Rutin) at 1%, 41 (Tropinone citrate) and 33 (Brucine) at 0.1% and 14 (Naringin) at 5% gave the shortest average development time and least eggs. Chemical 27 (Cinnamic acid) provided short average development time and a large number of eggs, but the bean control (2) gave the largest number of eggs overall and with fastest average development.

The four corners of this plot can be investigated for interesting chemicals.

Top left: bad for eggs, bad for development;

Top right: good for eggs, bad for development;

Bottom left: bad for eggs, good for development;

Bottom right: good for eggs, good for development.

The situation for minimum development time largely reflects that of average development time. Chemicals 12 (Vanillin) at 5% and 41 (Tropinone citrate) at 1% gave long minimum development time with low numbers of eggs. Chemicals 4 (L-Canavanine) at 1% and 34 (Strychnine) at 0.1% gave long minimum development time with greater numbers of eggs. Chemicals 20 (Homoprotocatehuic acid), 30 (Rutin) and 31 (Salicine) also gave long minimum development time with relatively large numbers of eggs. Chemicals 17 (Gallic acid), 23 (Digitonine), 32 (Isatin) and 41 (Tropinone citrate) at 0.1%, 10 (Syringic acid) and 30 (Rutin) at 1% and 14 (Naringin) at 5% gave quickest minimum development time but with low numbers of eggs. Chemical 27 (Cinnamic acid) gave relatively quick minimum development with a large number of eggs and the bean control (2) gave the largest number of eggs overall combined with quick minimum development time.

.

As expected, the influence of examined chemicals on development time was similar between the sexes as there was little difference between males and females for average development time, with the picture for both sexes reflecting the overall picture for average development time already seen. However, it is notable that chemicals 31 (Salicine) at 5% and 34 (Strychnine) at 0.1% gave considerably longer average development time for males than for females.
