# Supplementary material for: A semiochemical view of the ecology of the seed beetle Acanthoscelides obtectus Say (Coleoptera: Chrysomelidae, Bruchinae)
Source: Ann Appl Biol. 2023 Sep 4;184(1):19–36. doi: 10.1111/aab.12862 (PMC10953445; doi:10.1111/aab.12862)
Supplement: Supplementary file 2 — Data S2. Supporting information. [file AAB-184-19-s005.docx]

**A semiochemical view of the ecology of the seed beetle *Acanthoscelides obtectus* Say (Coleoptera: Chrysomelidae, Bruchinae)**

József Vuts, Stephen J Powers, Eudri Venter, Árpád Szentesi

Acceptable non-host^1^ legumes supporting larval development to adults of the seed beetle (A. obtectus) to various extent^2^ and possessing naturally occurring secondary plant substances (SPSs) in seeds

| Plant tribe and species^3^ | Characteristic SPSs in seeds | Reference |
| --- | --- | --- |
|  |  |  |
| **Genisteae** |  |  |
| *Lupius albus* L. | quinolizidine alkaloids (lupanine); storage proteins (globulins, legumin, vicilin); phenols (e.g., aesculin) | 1, 2, 3, 4, 5, 6 |
| **Phaseoleae** |  |  |
| *Glycine max* (L.) Merr. | storage proteins (legumin); triterpene alcohols (e.g., beta-amyrin); soyasaponins; gallic acid | 5, 7, 8, 9, 10, 11 |
| *Lablab purpureus* (L.) Sweet | polyamines; seed proteins (trypsin and chymotrypsin inhibitors); storage proteins (arcelin); lectins; lablab saponin; steroids; alkaloids | 6, 12, 13, 14, 15, 16 |
| *Vigna unguiculata* (L.) Walp. | storage proteins (vicilin); condensed tannins; fatty acids; flavon glycoside (vitexin); flavonoids; alkaloids; steroids | 6, 17, 18, 19, 20, 21 |
| *Vigna angularis* [(Willd.) Ohwi & H. Ohashi](https://ildis.org/cgi-bin/Araneus.pl?version~10.01&LegumeWeb&tno~2196&genus~Vigna&species~angularis) | triterpenoid saponins; quercetin and kaempferol glycosides; flavonoids; antocyanins; alkaloids; non-protein amino acids | 6, 21, 22, 23 |
| *Vigna radiata* [(L.) R. Wilczek](https://ildis.org/cgi-bin/Araneus.pl?version~10.01&LegumeWeb&tno~2258&genus~Vigna&species~radiata) | phenols (e.g., umbelliferon); seed proteins (trypsin and chymotrypsin inhibitors); condensed tannins; cyclopeptid alkaloid; terpenoids; steroids; alkaloids | 5, 6, 13, 24, 25 |
| *Phaseolus vulgaris* L. | saponins; flavonoids; phenols (e.g., aesculin); storage proteins (phaseolin, arcelin); glycoside; seed lectin (phytohemagglutinin); tannin; steroids; alkaloids; terpenoids | 5, 6, 10, 23, 26, 27, 28, 29, 30, 31, 32, 33, 34 |
| *Phaseolus coccineus* L. | glycosides; saponins; terpenoids; alkaloids; flavonoids | 6, 23, 35, 36, 37 |
| **Cicereae** |  |  |
| *Cicer arietinum* L. | triterpoene alcohols; oxo-sterols (stigmastenone); saponins; flavonoids; phenols (e.g., umbelliferon); seed proteins (trypsin and chymotrypsin inhibitors);  lectins; terpenoids; alkaloids | 5, 6, 8, 10, 13, 38 |
| **Fabeae** |  |  |
| *Vicia faba* L. | glycoside (vicine); condensed tannin; flavones; sterols; non-protein amino acids (DOPA); polyphenols; alkaloids | 6, 39, 40, 41, 42, 43, 44, 45, 46 |
| *Lens culinaris* Medik. | oxo-streroids; gallic acid; triterpene alcohols (e.g., amyrin); sterols; saponins; polyphenols (luteolin, kaempferol glycoside, etc.); imidazole; alkaloids; flavonoids | 5, 6, 8, 47, 48, 49, 50 |
| *Lathyrus hirsutus* L. | non-protein amino acids | 6 |
| *Lathyrus latifolius* L. | non-protein amino acids | 5, 6 |
| *Lathyrus odoratus* L. | non-protein amino acids; alkaloids | 6, 43 |
| *Lathyrus pratensis* L. | non-protein amino acids | 6 |
| *Lathyrus sativus* L. | triterpene alcohols; sterols; non-protein amino acids; phenolics (condensed tannins); alkaloids; flavonoids | 6, 8, 47, 51, 52, 53, 54, 55 |
| *Lathyrus tuberosus* L. | non-protein amino acids | 6 |
| *Pisum sativum* L. | storage proteins (globulins, legumin); triterpoene alcohols; oxo-sterols (stigmastenone); isoflavone phytoalexin (pisatin); saponins; alkaloids; condensed tannins | 2, 6, 8, 54, 56, 57, 58, 59 |

^1^de Boer and Hanson (1984), ^2^Szentesi (2021), ^3^Species and authority names are given according to ILDIS (International Legume Database & Information Service) <https://ildis.org/index.shtml>

References within table

(1) Mohamed, M. H., et al. (1994). (+)-15á-hydroxy-17-oxolupanine, a lupin alkaloid from the seeds of *Lupinus albus*. Phytochemistry 37(6): 1751-1754.

(2) Adler, L. S., et al. (2001). Direct and indirect effects of alkaloids on plant fitness via herbivory and pollination. Ecology 82(7): 2032-2044.

(3) Melo, T. S., et al. (1994). The seed storage proteins from *Lupinus albus*. Phytochemistry 37(3): 641-648.

(4) Muzquiz, M., et al. (1994). Variation of alkaloid components of lupin seeds in 49 genotypes of *Lupinus albus* L. from different countries and locations. Journal of Agricultural and Food Chemistry 42(7): 1447-1450.

(5) PubChem, National Library of Medicine. [https://pubchem.ncbi.nlm.nih.gov](https://pubchem.ncbi.nlm.nih.gov/compound/2244)

(6) Bisby, F. A. et al. (1994). Phytochemical Dictionary of the Leguminosae. Vols. 1 & 2. Dordrecht, Chapman & Hall/Springer-Science+Business Media, B.V.

(7) Coates, J. B., et al. (1985). Characterization of the subunits of β-conglycinin. Archives of Biochemistry and Biophysics 243(1): 184-194.

(8) Akihisa, T., et al. (1994). Triterpene alcohols and 3-oxo steroids on nine leguminosae seeds. Phytochemistry 35(5): 1309-1313.

(9) Connolly, J. D. and R. A. Hill (1985). Triterpenoids. Natural Product Reports 2: 421-441.

(10) Fenwick, D. E. and D. Oakenfull (1983). Saponin content of food plants and some prepared foods. Journal of the Science of Food and Agriculture 34(2): 186-191.

(11) Miyao, H., et al. (1996). Triterpene saponins from *Abrus cantoniensis* (Leguminosae). I. Isolation and characterization of four new saponins and a new sapogenol. Chemical & Pharmaceutical Bulletin 44(6): 1222-1227.

(12) Hamana, K., et al. (1992). Aminopropylaminoalcohols in the seeds of *Dolichos lablab*. Phytochemistry 31(3): 893-894.

(13) Ignacimuthu, S., et al. (2000). Chemical basis of resistance in pulses to *Callosobruchus maculatus* (F.) (Coleoptera: Bruchidae). Journal of Stored Products Research 36(1): 89-99.

(14) Janarthanan, S., et al. (2008). Arcelins from an Indian wild puls, *Lablab purpureus*, and insecticidal activity in storage pests. Journal of Agricultural and Food Chemistry 56(5): 1676-1682.

(15) Leopoldo, P. D. G., et al. (1994). Lectins of *Lablab purpureus* seeds. Journal of the Science of Food and Agriculture 65(2): 179-184.

(16) Yoshiki, Y., et al. (1995). A saponin conjugated with 2,3-dihydro-2,5-dihydroxy-6-methyl-4H-pyran-4one from *Dolichos lablab*. Phytochemistry 38(1): 229-232.

(17) Gomes, V. M., et al. (1997). Vicilin storage proteins from *Vigna unguiculata* (legume) seeds inhibit fungal growth. Journal of Agricultural and Food Chemistry 45(10): 4110-4115.

(18) Lattanzio, V., et al. (2005). Seed coat tannins and bruchid resistance in stored cowpea seeds. Journal of the Science of Food and Agriculture 85(5): 839-846.

(19) Piergiovanni, A. R., et al. (1990). Fatty acid composition and insect resistance in *Vigna unguiculata* seeds. Journal of the Science of Food and Agriculture 52(1): 47-53.

(20) Price, M. L., et al. (1980). Tannin content of cowpeas, chickpeas, pigeon peas, and mung beans. Journal of Agricultural and Food Chemistry 28(2): 459-461.

(21) Seneviratne, G. I. and J. B. Harborne (1992). Constitutive flavonoids and induced isoflavonoids as taxonomic markers in the genus *Vigna*. Biochemical Systematics and Ecology 20(5): 459-468.

(22) Iida, T., et al. (1999). Triterpenoid saponins from *Vigna angularis*. Phytochemistry 551(8): 1055-1058.

(23) Yoshida, K., et al. (1996). Structural analysis and measurement of anthocyanins from colored seed coats of *Vigna*, *Phaseolus*, and *Glycine* legumes. Bioscience, Biotechnology and Biochemistry 60(4): 589-593.

(24) Barroga, C. F., et al. (1985). Effect of condensed tannins on the in vitro protein digestibility of mungbean (*Vigna radiata* (L.) Wilczek). Journal of Agricultural and Food Chemistry 33(6): 1157-1159.

(25) Sugawara, F., et al. (1996). Insecticidal peptide from mungbean: A resistant factor against infestation with azuki bean weevil. Journal of Agricultural and Food Chemistry 44(10): 3360-3364.

(26) Baldin, E. L. L. and F. M. Lara (2008). Resistance of stored bean varieties to *Acanthoscelides obtectus* (Coleoptera : Bruchidae). Insect Science 15(4): 317-326.

(27) Lioi, L., et al. (2003). Lectin-related resistance factors against bruchids evolved through a number of duplication events. Theoretical and Applied Genetics 107(5): 814-822.

(28) Mirkov, T. E., et al. (1994). Evolutionary relationships among proteins in the phytohemagglutinin-arcelin-alpha-amylase inhibitor family of the common bean and its relatives. Plant Molecular Biology 26(4): 1103-1113.

(29) Osborn, T. C., et al. (1988). Insecticidal activity and lectin homology of arcelin seed protein. Science 240(4849): 207-210.

(30) Paes, N. S., et al. (2000). The effect of arcelin-1 on the structure of the midgut of bruchid larvae and immunolocalization of the arcelin protein. Journal of Insect Physiology 46(4): 393-402.

(31) Ma, Y. and F. A. Bliss (1978). Tannin content and inheritance in common bean. Crop Science 18(2): 201-204.

(32) Silva, L. B., et al. (2004). The seed coat of *Phaseolus vulgaris* interferes with the development of the cowpea weevil [*Callosobruchus maculatus* (F.) (Coleoptera: Bruchidae)]. Anais da Academia Brasileira de Ciências 76(1): 57-65.

(33) Akihisa, T., et al. (1989). 25-Methylgramisterol and other 4α-methylsterols from *Phaseolus vulgaris* seeds. Phytochemistry 28(4): 1219-1224.

(34) Fernández, R., et al. (1982). Trypsin inhibitors and hemagglutinins in beans (*Phaseolus vulgaris*) and their relationship with the content of tannins and associated polyphenols. Journal of Agricultural and Food Chemistry 30(4): 734-739.

(35) Yoshiki, Y., et al. (1994). Saponins conjugated with 2,3-dihydro-2,5-dihydroxy-6-methyl-4H-pyran-4-one from *Phaseolus coccineus*. Phytochemistry 36(4): 1009-1012.

(36) Schliemann, W., et al. (1994). Native gibberellin-O-glucosides from nature seeds of *Phaseolus coccineus*. Phytochemistry 35(1): 35-38.

(37) Adesanya, S. A., et al. (1985). Isoflavonoids from *Phaseolus coccineus*. Phytochemistry 24(11): 2699-2702.

(38) Zhankui, W., et al. (2017). Isolation, identification and characterization of a new type of lectin with alpha-amylase inhibitory activity in chickpea (*Cicer arietinum* L.). Protein & Peptide Letters 24(11): 1008-1020.

(39) Desroches, P., et al. (1997). Mediation of host-plant use by a glucoside in *Callosobruchus maculatus* F (Coleoptera: Bruchidae). Journal of Insect Physiology 43(5): 439-446.

(40) Martin-Tanguy, J., et al. (1977). Condensed tannins in horse bean seeds: Chemical structure and apparent effects on poultry. Journal of the Science of Food and Agriculture 28(8): 757-765.

(41) Bekkara, F., et al. (1998). Distribution of phenolic compounds within seed and seedlings of two *Vicia faba* cvs differing in their seed tannin content, and study of their seed and root phenolic exudations. Plant and Soil 203(1): 27-36.

(42) Cerri, R., et al. (1985). Sterols, steryl esters and fatty acids in some *Vicia faba* seeds. Biochemical Systematics and Ecology 13(1): 11-13.

(43) Fowden, L. (1970). The non-protein amino acids of plants. Progress in Phytochemistry. L. Reinhold and Y. Liwschitz. London, etc., Interscience Publishers. 2: 203-266.

(44) Griffiths, D. W. (1981). The polyphenolic content and enzyme inhibitory activity of testas from bean (*Vicia faba*) and pea (*Pisum* spp.) varieties. Journal of the Science of Food and Agriculture 32(8): 797-804.

(45) Hebblethwaite, P. D., Ed. (1983). The Faba Bean (*Vicia faba* L.). A Basis for Improvement. London, etc., Butterworths.

(46) Torck, M. and M. Pinkas (1992). Les flavonoides du genre *Vicia*. Biochemical Systematics and Ecology 20(5): 453-457.

(47) Akihisa, T., et al. (1991). Sterols of three leguminosae seeds: occurrence of 24α-ethyl-5α-cholest-9(11)-en-3β-ol and both c-24 epimers of 24-ethylcholesta-5,25-dien-3β-ol. Phytochemistry 30(12): 4029-4032.

(48) Applebaum, S. W., et al. (1969). Saponins as possible factors of resistance of legume seeds to the attack of insects. Journal of Agricultural and Food Chemistry 17(3): 618-622.

(49) D'Arcy, A. and M. Jay (1978). Les flavonoïdes des graines de *Lens culinaris*. Phytochemistry 17(4): 826-827.

(50) Hayman, A. R. and D. O. Gray (1987). Imidazole, a new natural product from the Leguminosae. Phytochemistry 26(12): 3247-3248.

(51) Fikre, A., et al. (2008). The contents of the neuro-excitatory amino acid β-ODAP (β-N-oxalyl-l-α,β-diaminopropionic acid), and other free and protein amino acids in the seeds of different genotypes of grass pea (*Lathyrus sativus* L.). Food Chemistry 110(2): 422-427.

(52) Aletor, V. A., et al. (1994). Evaluation of the seeds of selected lines of three *Lathyrus* spp. for á-N-oxalylamino-L-alanine (BOAA), tannins, trypsin inhibitor activity and certain in-vitro characteristics. Journal of the Science of Food and Agriculture 65(2): 143-152.

(53) Arentoft, A. M. K. and B. N. Greirson (1995). Analysis of 3-(N-oxalyl)-L-2,3-diaminopropanoic acid and its alfa-isomer in grass pea (*Lathyrus sativus*) by capillary zone electrophoresis. Journal of Agricultural and Food Chemistry 43(4): 935-941.

(54) Wang, X. F., et al. (1998). Total phenolics and condensed tannins in field pea (Pisum sativum L.) and grass pea (*Lathyrus sativus* L.). Euphytica 101(1): 97-102.

(55) Yan, Z.-Y., et al. (2006). *Lathyrus sativus* (grass pea) and its neurotoxin ODAP. Phytochemistry 67(2): 107-121.

(56) Matta, N. K., et al. (1981). Molecular and subunit heterogeneity of legumin of *Pisum sativum* l. (garden pea)— a multi-dimensional gel electrophoretic study. Journal of Experimental Botany 32(6): 1295-1307.

(57) Cooper, L. D., et al. (2005). Application of bruchin B to pea pods results in the up-regulation of CYP93C18, a putative isoflavone synthase gene, and an increase in the level of pisatin, an isoflavone phytoalexin. Journal of Experimental Botany 56(414): 1229-1237.

(58) 754 - Kobayashi, A., et al. (1993). A pterocarpan, (+)-2-hydroxypisatin from *Pisum sativum*. Phytochemistry 32(1): 77-78.

(59) Tsurumi, S., et al. (1992). A gamma-pyronyl-triterpenoid saponin from *Pisum sativum*. Phytochemistry 31(7): 2435-2438.
