# Supplementary material for: A semiochemical view of the ecology of the seed beetle Acanthoscelides obtectus Say (Coleoptera: Chrysomelidae, Bruchinae)
Source: Ann Appl Biol. 2023 Sep 4;184(1):19–36. doi: 10.1111/aab.12862 (PMC10953445; doi:10.1111/aab.12862)
Supplement: Supplementary file 3 — Data S3. Supporting information. [file AAB-184-19-s001.docx]

A semiochemical view of the ecology of the seed beetle *Acanthoscelides obtectus* Say (Coleoptera: Chrysomelidae, Bruchinae)

József Vuts, Stephen J Powers, Eudri Venter, Árpád Szentesi

**Coupled gas chromatography-electroantennography (GC-EAG)**

Antennal recordings were made using Ag–AgCl glass electrodes filled with saline solution composed as in Maddrell (1969), but without the glucose (7.55 g/L sodium chloride, 0.64 g/L potassium chloride, 0.22 g/L calcium chloride, 1.73 g/L magnesium chloride, 0.86 g/L sodium bicarbonate, 0.61 g/L sodium orthophosphate). An antenna was freshly amputated at the base from a live *A*. *obtectus* and suspended between the two electrodes. The tip of the terminal process of the antenna was removed to ensure a good contact with a high-impedance amplifier (UN-06; Ockenfels Syntech GmbH, Kirchzarten, Germany). The base of the antenna was connected to a grounded electrode. Separation of the collected male *A. obtectus* volatiles was achieved on a high-resolution gas chromatograph (Agilent 6890 N; Agilent Technologies, Santa Clara, CA, USA), equipped with a cool on-column injector and a flame ionization detector (FID) using a 50 m × 0.32 mm ID, 0.52 μm film thickness HP-1 column (J & W Scientific, Folsom, CA, USA). The oven temperature was maintained at 30°C for 2 min and then programmed at 15°C/min to 250°C. The carrier gas was helium. One μL aliquots of headspace extract were injected into the GC. The outputs from the EAG amplifier and the FID were monitored simultaneously and analysed using a customized software package (Syntech GC/EAD for Windows v 2.3 09/1997).

**Four-arm olfactometer assays**

The olfactometer consisted of three layers of Perspex, held together with plastic nuts and bolts. Both the top and bottom discs had a 156 mm diameter and 5 mm thickness, and the bottom disc was fitted with a filter paper base to provide traction for the walking insect. The middle part was 180 mm in diameter and 7 mm thick and was manufactured to embody four side areas or arms (55 mm in length × 5 mm height each) situated at 90° to each other. The side areas narrowed towards the perimeter and were directly connected via a 3 mm diameter hole at the end to glass arms (narrow part: 50 mm length × 2.5 mm diam., wide part: 90 mm length × 20 mm diam.). Prior to each experiment, all glassware was washed with Teepol (Orpington, UK) detergent, rinsed with acetone and distilled water and baked in an oven overnight at 160˚C. Perspex components were washed with Teepol solution, rinsed with 80% ethanol solution and distilled water and left to air-dry. The olfactometer was illuminated from above by diffuse uniform lighting from two 18W/35 white fluorescent light bulbs screened with red acetate. It was surrounded by black paper to remove any external visual stimuli. Test compounds were applied onto filter paper strips (ca. 2 cm2, Whatman, Little Chalfont, UK) in proportions and doses in such a way that the amounts released per hour were similar to those emitted by one male beetle over 1 h. As only one blend was tested, three control arms were treated with 10 μL redistilled hexane. After allowing the solvent to evaporate for 30 s, the strips were inserted into the glass arms. This setup ensured the robustness of the experiment by making it less likely for an insect to accidentally walk in or out of the treated region. A single beetle was introduced through a hole in the top of the olfactometer. Air was drawn through the central hole by a vacuum pump and, consequently, pulled through each of the four side arms (75 mL/min/arm) and subsequently exhausted from the room. Each beetle was given 2 min to acclimatize in the olfactometer (the room temperature was 20˚C and RH 60%), after which the experiment was run for 16 min. The olfactometer was rotated 90˚ every 4 min to control for any directional bias. The olfactometer was divided into four regions, corresponding to each of the four arms and the central compartment, and the time spent (min) in each arm was recorded using specialist software (OLFA, Udine, Italy). The structure of the experiment was such that there were two blend treatments (including control) tested in each one, giving an unbalance that precludes use of ANOVA. In order to account for the replication and areas within each replication as variance components in a split-plot design, the method of residual maximum likelihood (REML) was used to fit a linear mixed model to the time spent data, nesting the areas within each replication and testing the treatment effect using an approximate *F*-test. The data were analysed on the square root scale to account for some heterogeneity of variance over the treatments. Predicted means for statistically relevant (p *<* 0.05, F-test) terms from the model were output for comparison using Fisher’s LSD test (p *<* 0.05). Genstat was used for this analysis.

**Petri dish arena choice assays**

Petri dishes (55 mm diam., 13 mm height) with a 55 mm diam. filter paper on the bottom served as test arenas. Two pencil marks, 35 mm apart on the filter paper, indicated the places for the test (t) and control (c) female individuals (3–8-day-old). These were freeze-killed on dry ice before use in experiments and were laid on their side. If a beetle was soaked in hexane, the solvent was allowed to evaporate prior to testing. One male beetle (3–11-day-old) was put in each Petri dish arena (representing 1 replication), and the number of copulation attempts (mounting and penis extruded) toward the test and control freeze-killed individuals was recorded. Beetles were observed for 20 min (n=7-10).

Tests 1 and 4 were carried out to confirm earlier observations on male mating preference for female conspecifics. Freeze-killed virgin or mated *Zabrotes subfasciatus* females (t) and males (c) were compared. Test 2 investigated if the mounting and copulation-initiating cues are chemicals found on the surface of females. Virgin freeze-killed females (t) were tested against females soaked in hexane for 10 min (c). Test 3 assessed the effect of treating hexane-washed females with female extract (t). Hexane-washed females served as controls (c). Test females were coated with 2 μL (*c*. one female equivalent) of a hexane extract of 20 females using glass micropipettes (Brand GmbH, Germany), by evenly spreading the extract on the entire dorsal surface of an insect. A final test compared responses of male *Z. subfasciatus* and *A. obtectus* to con- and heterospecific female dummies.

To analyse the effect of treatment in each behavioural experiment separately, the difference in count was taken for each of the ten replicate arenas. These values were modelled assuming a Poisson distribution with a log-link function, fitting a generalized linear model (McCullagh and Nelder 1989) of the form log *(count_i_) = μ*, where *μ* is a constant and i=1,…10. We tested whether the mean difference in counts was different (at α=0.05) from 0, on 9° of freedom (being the number of arenas used less 1). The predicted mean difference was then output with appropriate standard error.

**Scanning Electron Microscopy**

*A. obtectus* beetles and ovipositors were supplied in 70% ethanol. Beetles/ovipositors were removed from 70% ethanol, rinsed quickly with distilled H_2_O, transferred onto SEM stubs (whole beetles mounted upside down to easily image the ovipositors) coated with a 1:1 mixture of Graphene Oxide (TAAB) and Tissue Tek (Sakura). CryoSEM sample preparation was done with a Quorum PP3010T sample preparation device (Quorumtech, UK). Briefly, samples were plunged frozen into a slush of liquid nitrogen, transferred to the cryo prep chamber under vacuum, coated with Platinum for 2 min at 5uA. SEM imaging was done on a JEOL JSM-6360LV scanning electron microscope (JEOL Ltd.), with imaging conditions 5kV, 30 spot size and 12-15 mm working distance. Imaging freshly protruded ovipositor from live beetles was unsuccessful due to the amount of body fluid covering the ovipositors.
