# Supplementary material for: A semiochemical view of the ecology of the seed beetle Acanthoscelides obtectus Say (Coleoptera: Chrysomelidae, Bruchinae)
Source: Ann Appl Biol. 2023 Sep 4;184(1):19–36. doi: 10.1111/aab.12862 (PMC10953445; doi:10.1111/aab.12862)
Supplement: Supplementary file 4 — Data S4. Supporting information. [file AAB-184-19-s003.docx]

A semiochemical view of the ecology of the seed beetle *Acanthoscelides obtectus* Say (Coleoptera: Chrysomelidae, Bruchinae)

József Vuts, Stephen J Powers, Eudri Venter, Árpád Szentesi

**Multiple Choice Experiment**

**Data and Methods**

There were 42 compounds and two controls to be compared as regards fecundity of the bean beetle. They were grouped into 27 classes with some compounds in singleton classes. The compounds were (* occurring in *Fabaceae*):

**No. Compound/Control Class**

1 L-canavanine* Nonprotaa

2 Hordenin sulphate* Alk

3 Glass bead (8 mm diam) Glass bead control

4 Condensed tannin Polyflavonoid

5 Tannin Polyphenol

6 Picrotoxin Pycrotoxin

7 Quinidine Alk

8 Syringic acid Bensoic acid

9 Smilagenine Steroid saponin

10 Vanillin Phenolic aldehyde

11 Theophylline Alk

12 Naringin Flavonoid

13 Eserine* Alk

14 Morin Flavonoid

15 Gallic acid Phenolic acid

16 Coumarine Chromen

17 Arbutin Glycoside

18 Homoprotocatechuic acid Phenyl acetate

19 Aesculine Phenolic glycoside

20 Caffeine Methylxanthine

21 Digitonin Cardenolide

22 Barbitural Barbitural

23 Reserpine Alk

24 Hecogenin Steroid sapogenine

25 Cinnamic acid Cinnamic acid

26 Umbelliferon Hydroxycoumarine

27 Tomatine Glycoalkaloid

28 Rutin Flavonoid

29 Salicin phenolic Glycoside

30 Isatin Indol derivative

31 Brucine Alk

32 Strychnine Alk

33 Quinine Alk

34 Ergotamin Alk

35 Codeine Alk

36 Aconitic acid Organic acid

37 Nicotine H-tartrate* Alk

38 Sodium oxalate Organic acid

39 Tropinone Alk

40 Atropine Alk

41 Genistein Isoflavonoid

42 Solasodine Steroid glycoalk

43 Vincamin Alk

44 Cotyledon pilule Cotyledon pilule control

Three devices with hexagonal positions (sections) were used for the experiment. The hexagonal positions contained artificial beans called pilules treated with the 42 compounds plus controls (Cotyledon-only pilule or Glass bead) to be compared. A maximum of 88 positions per device were filled. Hence, one device was used per replicate run, with two positions per compound per device. The design of the experiment can therefore be seen as a split-plot. All 42 compounds were tested at concentration 0.1 w/w% (plus control), 39 at 1 w/w% (excluding Eserine, Ergotamin and Solasodin) (plus controls) and 33 at 5 w/w% (excluding Condensed tannin, Smilagenine, Eserine, Barbitural, Reserpine, Hecogenin, Tomatine, Ergotamin and Vincamin) (plus controls). There were six replicate runs per substrate per concentration, each device being used twice within a concentration. One hundred females and 10 males were inserted into each device for each run and left for three days. Numbers of eggs laid in each position in a device were then counted. See **Appendix** for a table of the raw means and SEs.

A linear mixed model (LMM) was fitted to the natural logarithm of the count data, having added a small adjustment (0.5) to account for zero observations. This transformation allowed the data to conform to the assumptions of the analysis (a Normal distribution and constant variance over the treatments).

The model fitted was:

Log(*Count* + 0.5) ~ *Constant* + (*Class*/*Compound*)**Conc* + *Device*/*DeviceRun*/*Position*

where the ‘/’ indicates nesting of factors and ‘*’ indicates that main effects and interactions between factors were included. *Class*, *Compound* and *Conc* were the fixed effect terms in the model and *Device*, *DeviceRun* and *Position* were the random effect (design) terms, with runs of the devices (*DeviceRun*) being nested in *Device* and *Position* being nested in *DeviceRun*.

The significance of fixed effect terms was tested using Wald (Chi-squared) tests as part of the residual maximum likelihood (REML) method used for fitting the model. Means from statistically significant (p < 0.05, Chi-squared) terms were output for comparison of pairs of means of particular interest using approximate least significant difference (LSD) values at the 5% level of significance.

An alternative analysis is to assume a Poisson distribution for the counts and fit a generalised linear mixed model (GLMM), but as there weren't overly many zeros, residual plots confirmed that a Normal distribution for data on the log-scale could be assumed.

In order to investigate and compare the compounds visually, principal component analysis (PCA) was applied to the data for each concentration separately, using the sums of the two counts per compound, device and runs within devices as the variables and the compounds themselves as the units. The analysis was based on the correlation matrix.

Cluster analysis was also applied to the data, based on the Euclidean distance matrix between the profiles for the compounds given the total counts over the pseudo-replicates per compound, device and runs within devices as the variables. For the clustering, the group average method was used.

The Genstat (19th edition, VSN International Ltd, Hemel Hempstead, UK) statistical package was used for the analysis.

**Results**

There was a three-way interaction between compounds, nested in class, and concentration (p < 0.001, Chi-squared). This shows that the number of eggs laid varied over the chemicals within classes, and with increasing concentrations in a non-independent way. Splitting the grouped compounds from the singleton-group compounds, two charts were drawn.

**Note: Alks in grey, flavonoids in pink, organic acids in orange and phenolic glycosides in green. LSD_1_ (5%) (0.581) for comparison between the controls; LSD_2_ (5%) (0.858) for comparisons to either control; LSD_3_ (5%) (1.007) for comparisons within a concentration; LSD_4_ (5%) (1.093) for all other comparisons, on 604 df.**

*** Combination not assessed due to insufficient compound.**

Of the alks, Tropinone and Nicotine H-tartrate gave the most notable positive response as regards increasing numbers of eggs laid with increasing concentration. Of the flavonoids, Rutin gave a strong positive response. Of the organic acids, sodium oxalate gave a positive response at the greatest concentration and Aconitic acid gave a peaked response, with 1 w/w% providing maximal fecundity for this compound. Phenolic glycoside Aesculine had a curious inverted response, with higher fecundity at 0.1 and 5 w/w% than at 1 w/w% concentration.

**Note: LSD_1_ (5%) (0.581) for comparison between the controls; LSD_2_ (5%) (0.858) for comparisons to the cotyledon or glass bead control; LSD_3_ (5%) (1.007) for comparisons within a concentration; LSD_4_ (5%) (1.093) for all other comparisons, on 604 df. * Combination not assessed due to insufficient compound.**

The isoflavonoid Genistein gave a tremendous response at 5 w/w% concentration, as did the steroid saponin Smilagenine at 1 w/w% concentration and the cardenolide Digitonin at 5 w/w% concentration. The phenyl acetate Homoprotocatechuic acid and the methylxanthine Caffeine gave relatively strong positive responses with increasing concentration. The chromen Coumarine and the phenolic aldehyde Vanillin gave a decreasing response with very few eggs being laid, whereas the most dramatic negative response with increasing concentration was seen for Cinnamic acid.

The significance of fixed effects in the model is shown the table below.

Wald tests for fixed effects

----------------------------

Sequentially adding terms to fixed model

**Fixed term Wald statistic d.f. Wald/d.f. chi pr**

Class 340.50 26 13.10 <0.001

Conc 0.50 2 0.25 0.778

Class.Compound 178.35 17 10.49 <0.001

Class.Conc 127.07 42 3.03 <0.001

Class.Compound.Conc 77.40 28 2.76 <0.001

Principal components analysis (PCA) of the data at 0.1 w/w% concentration revealed some separation of the chemicals in the two dimensional plot of the first *versus* the second principal component (PC).


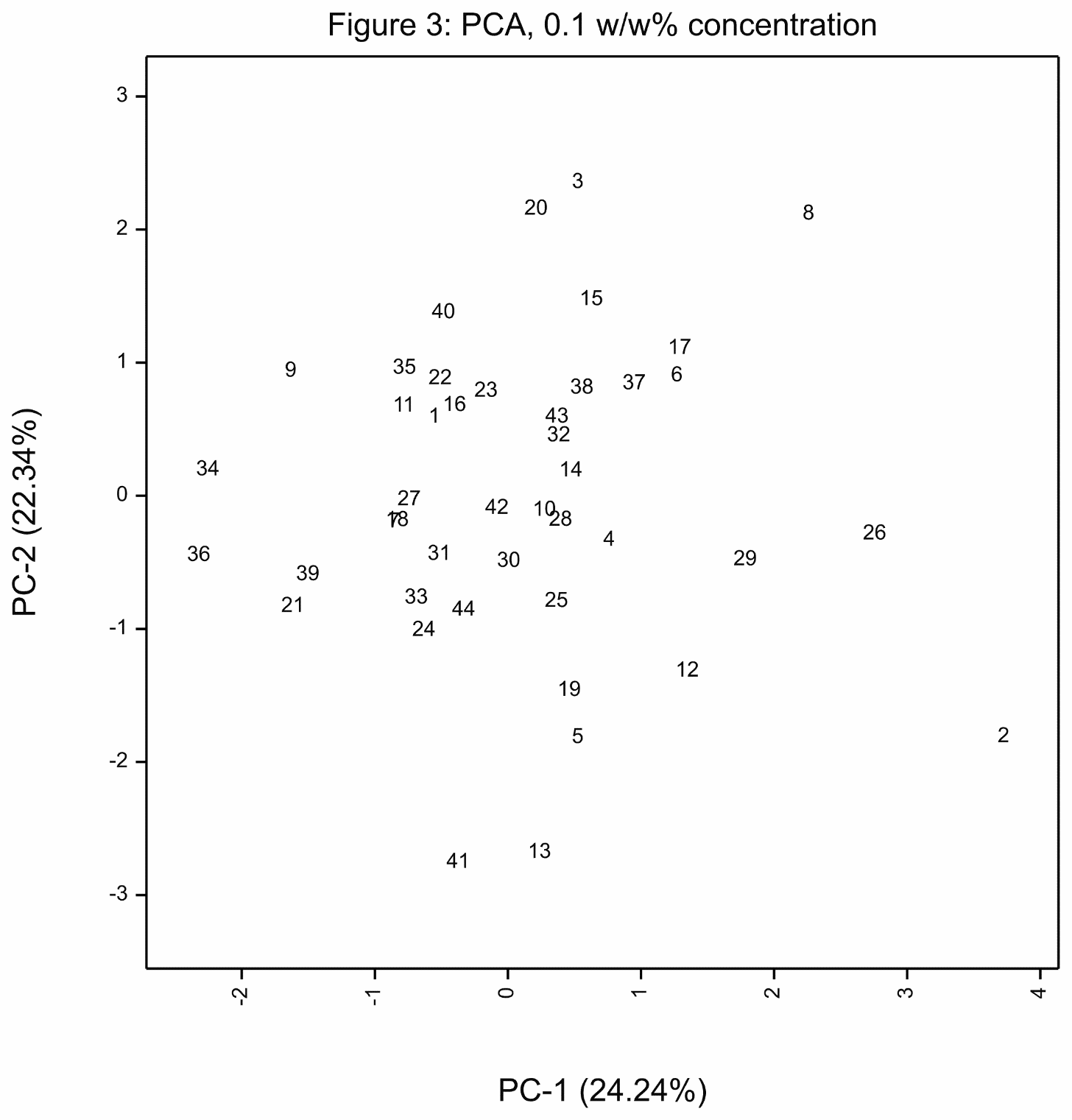


Note: for clarity numbers are used in the plot to represent the compounds (class): 1 L-canavanine* (Nonprotaa), 2 Hordenin sulphate* (Alk), 3 Glass bead, 4 Condensed tannin (Polyflavonoid), 5 Tannin (Polyphenol), 6 Pycrotoxin, 7 Quinidine (Alk), 8 Syringic acid (Bensoic acid), 9 Smilagenine (Steroid saponin), 10 Vanillin (Phenolic aldehyde), 11 Theophylline (Alk), 12 Naringin (Flavonoid), 13 Eserine* (Alk), 14 Morin (Flavonoid), 15 Gallic acid (Phenolic acid), 16 Coumarine (Chromen), 17 Arbutin (Glycoside), 18 Homoprotocatechuic acid (Phenyl acetate), 19 Aesculine (Phenolic glycoside), 20 Caffeine (Methylxanthine), 21 Digitonin (Cardenolide), 22 Barbitural, 23 Reserpine (Alk), 24 Hecogenin (Steroid sapogenine), 25 Cinnamic acid, 26 Umbelliferon (Hydroxycoumarine), 27 Tomatine (Glycoalkaloid), 28 Rutin (Flavonoid), 29 Salicin phenolic (Glycoside), 30 Isatin (Indol derivative), 31 Brucine (Alk), 32 Strychnine (Alk), 33 Quinine (Alk), 34 Ergotamin (Alk), 35 Codeine (Alk), 36 Aconitic acid (Organic acid), 37 Nicotine H-tartrate* (Alk), 38 Sodium oxalate (Organic acid), 39 Tropinone (Alk), 40 Atropine (Alk), 41 Genistein (Isoflavonoid), 42 Solasodine (Steroid glycoalk), 43 Vincamin (Alk), 44 Cotyledon pilule (Control) (* occurring in *Fabaceae*).

Interestingly, two of the compounds occurring in *Fabaceae*, Hordenin sulphate (2) and Eserine (13) were well-separated from the others in the plot, along with Genistein (41). Also, glass bead (3), Syringic acid (8) and Caffeine (20) were separated from the others on PC2, whereas Ergotamin (34) and Aconitic acid (36) were separated from the others on PC1.

Cluster analysis of the data revealed similar results, with Hordenin sulphate (2) being most dissimilar from the other compounds. Three main clusters of compounds, two clusters of two compounds (Eserine (13) and Genistein (41); Brucine (31) and Atropine (40)) and the singleton cluster (Hordenin sulphate (2)) may be formed at a similarity of around 0.88.


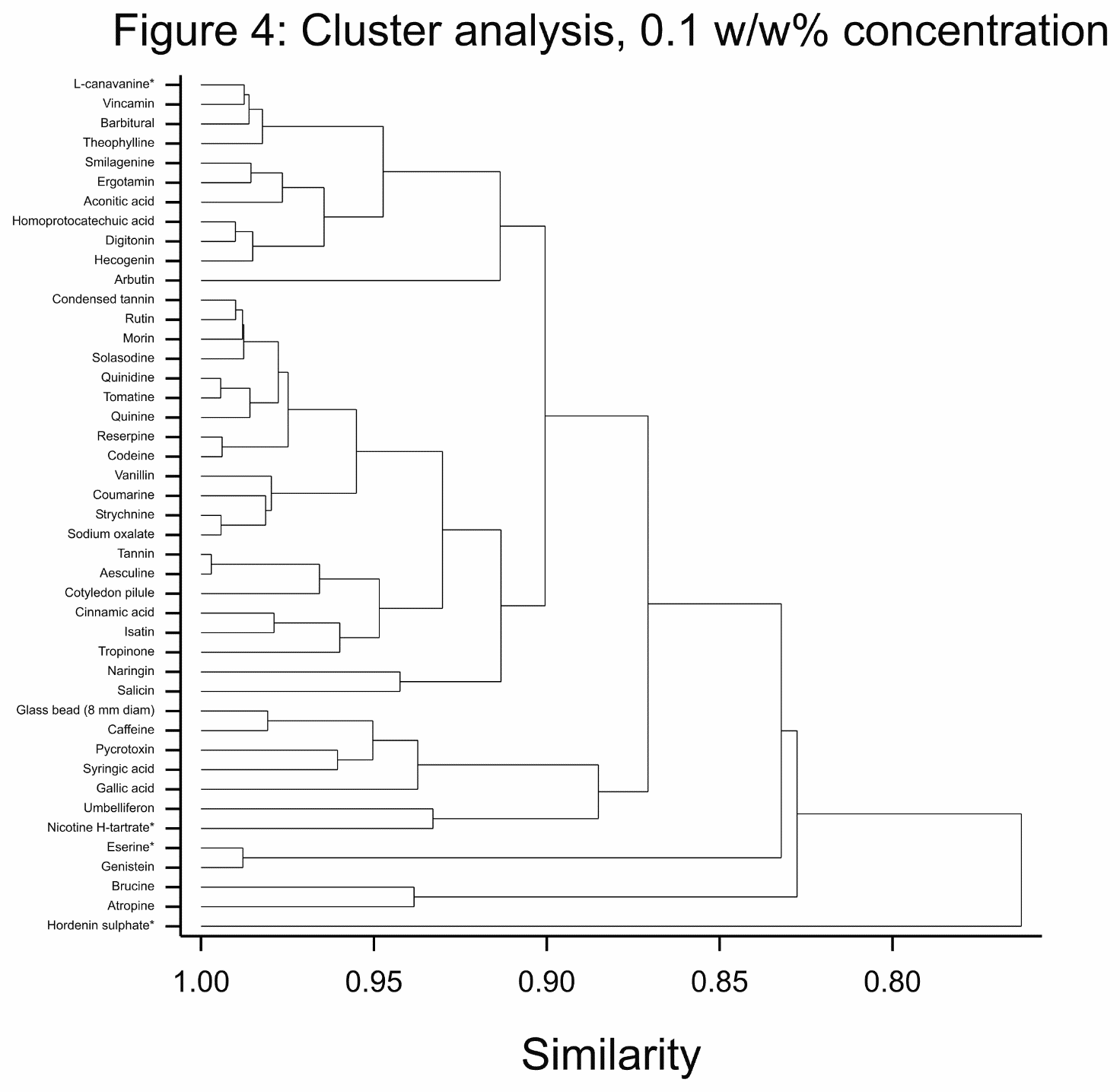


Principal components analysis (PCA) of the data at 1 w/w% concentration revealed an improved separation of the chemicals in the two dimensional plot of the first *versus* the second principal component (PC).


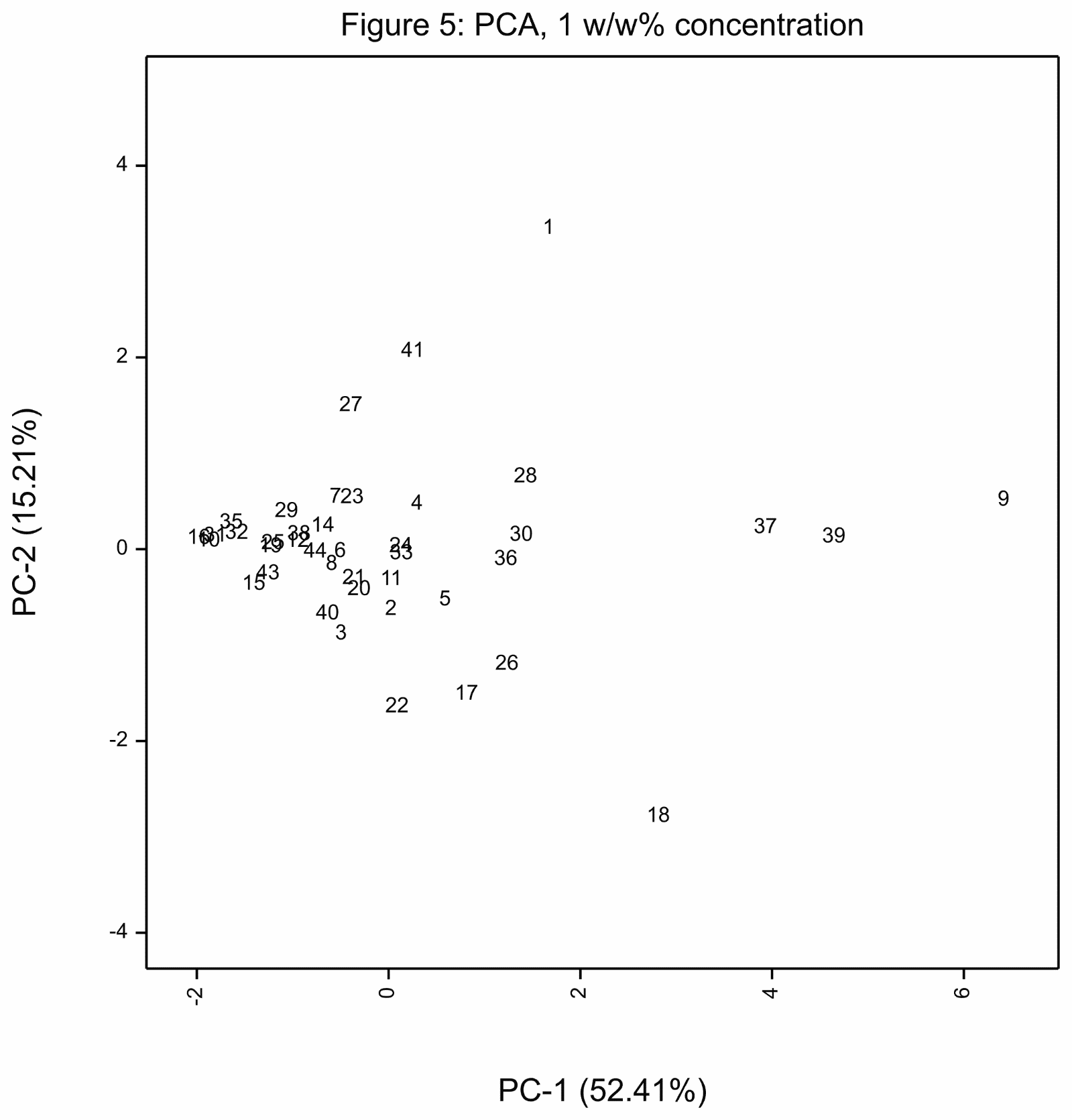


Note: for clarity numbers are used in the plot to represent the compounds (class): 1 L-canavanine* (Nonprotaa), 2 Hordenin sulphate* (Alk), 3 Glass bead, 4 Condensed tannin (Polyflavonoid), 5 Tannin (Polyphenol), 6 Pycrotoxin, 7 Quinidine (Alk), 8 Syringic acid (Bensoic acid), 9 Smilagenine (Steroid saponin), 10 Vanillin (Phenolic aldehyde), 11 Theophylline (Alk), 12 Naringin (Flavonoid), ~~13 Eserine* (Alk)~~, 14 Morin (Flavonoid), 15 Gallic acid (Phenolic acid), 16 Coumarine (Chromen), 17 Arbutin (Glycoside), 18 Homoprotocatechuic acid (Phenyl acetate), 19 Aesculine (Phenolic glycoside), 20 Caffeine (Methylxanthine), 21 Digitonin (Cardenolide), 22 Barbitural, 23 Reserpine (Alk), 24 Hecogenin (Steroid sapogenine), 25 Cinnamic acid, 26 Umbelliferon (Hydroxycoumarine), 27 Tomatine (Glycoalkaloid), 28 Rutin (Flavonoid), 29 Salicin phenolic (Glycoside), 30 Isatin (Indol derivative), 31 Brucine (Alk), 32 Strychnine (Alk), 33 Quinine (Alk), ~~34 Ergotamin (Alk)~~, 35 Codeine (Alk), 36 Aconitic acid (Organic acid), 37 Nicotine H-tartrate* (Alk), 38 Sodium oxalate (Organic acid), 39 Tropinone (Alk), 40 Atropine (Alk), 41 Genistein (Isoflavonoid), ~~42 Solasodine (Steroid glycoalk)~~, 43 Vincamin (Alk), 44 Cotyledon pilule (Control) (* occurring in *Fabaceae*). Crossed-out compounds are not represented at this concentration.

The control (cotyledon) is well-separated from the compounds, as are Smilagenine (a Steroid saponin) (9), Nicotine H-tartrate (an Alk) (37), Tropinone (an Alk) (39) and Homoprotocatechuic acid (a Phenyl acetate) (18).


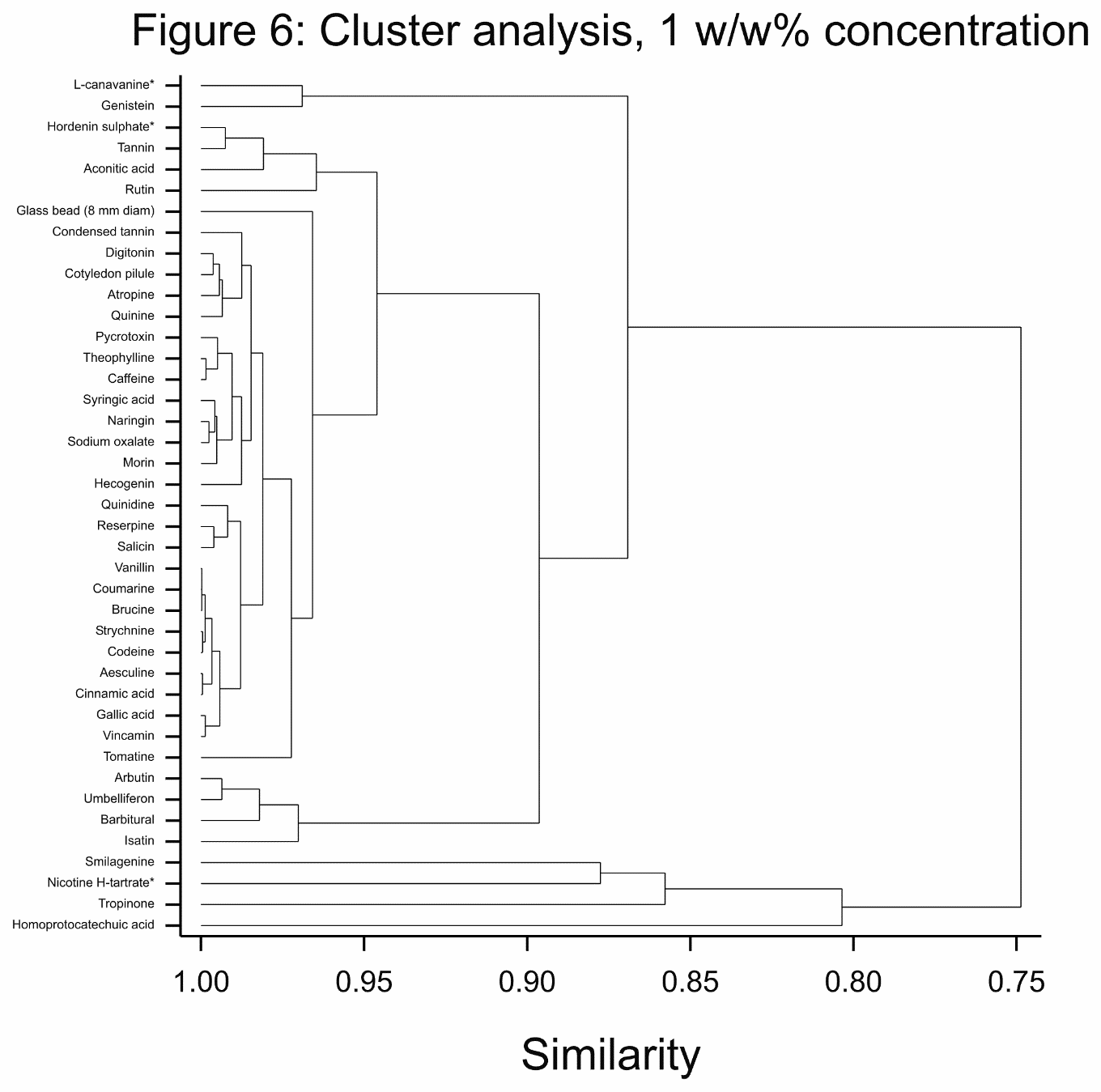


Working from the bottom upwards, at a similarity of 0.95, the cluster analysis shows four singletons (Homoprotocatechuic acid (Phenyl acetate), Tropinone (Alk), Nicotine H-tartrate (Alk) and Smilagenine (Steroid saponin), a cluster of four compounds (Arbutin (Glycoside), Umbelliferon (Hydroxycoumarine), Barbitural and Isatin (Indol derivative)), a main cluster of 31 compounds and a cluster of two compounds (L-canavanine (Nonprotaa) and Genistein (Isoflavonoid)).

Principal components analysis (PCA) of the data at 5 w/w% concentration showed separation of the chemicals in the two dimensional plot of the first *versus* the second principal component (PC), with almost 70% of the variance in the data being accounted for by the first PC.


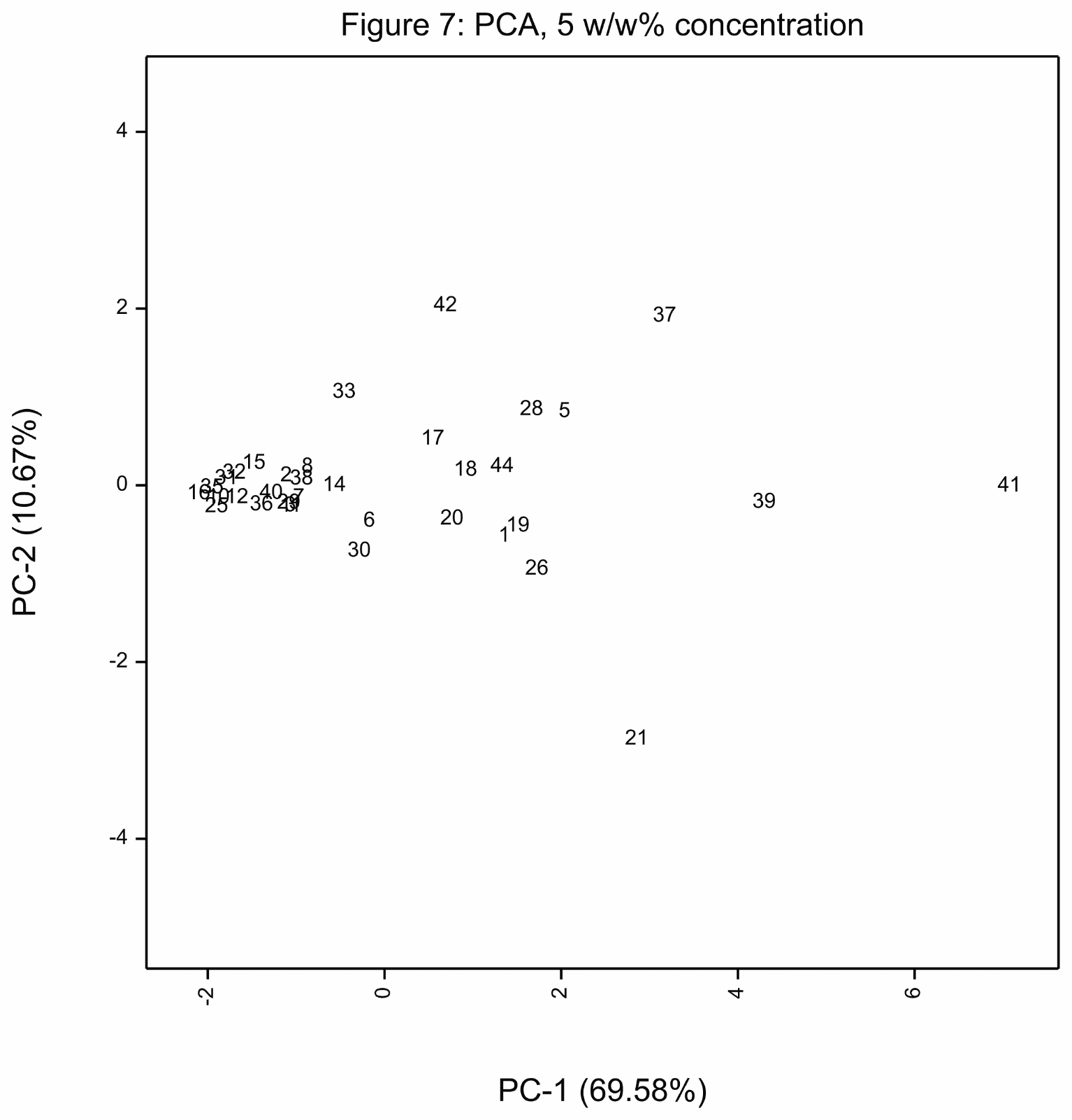


Note: for clarity numbers are used in the plot to represent the compounds (class): 1 L-canavanine* (Nonprotaa), 2 Hordenin sulphate* (Alk), 3 Glass bead, ~~4 Condensed tannin (Polyflavonoid)~~, 5 Tannin (Polyphenol), 6 Pycrotoxin, 7 Quinidine (Alk), 8 Syringic acid (Bensoic acid), ~~9 Smilagenine (Steroid saponin)~~, 10 Vanillin (Phenolic aldehyde), 11 Theophylline (Alk), 12 Naringin (Flavonoid), ~~13 Eserine* (Alk)~~, 14 Morin (Flavonoid), 15 Gallic acid (Phenolic acid), 16 Coumarine (Chromen), 17 Arbutin (Glycoside), 18 Homoprotocatechuic acid (Phenyl acetate), 19 Aesculine (Phenolic glycoside), 20 Caffeine (Methylxanthine), 21 Digitonin (Cardenolide), ~~22 Barbitural, 23 Reserpine (Alk), 24 Hecogenin (Steroid sapogenine)~~, 25 Cinnamic acid, 26 Umbelliferon (Hydroxycoumarine), ~~27 Tomatine (Glycoalkaloid)~~, 28 Rutin (Flavonoid), 29 Salicin phenolic (Glycoside), 30 Isatin (Indol derivative), 31 Brucine (Alk), 32 Strychnine (Alk), 33 Quinine (Alk), ~~34 Ergotamin (Alk)~~, 35 Codeine (Alk), 36 Aconitic acid (Organic acid), 37 Nicotine H-tartrate* (Alk), 38 Sodium oxalate (Organic acid), 39 Tropinone (Alk), 40 Atropine (Alk), 41 Genistein (Isoflavonoid), 42 Solasodine (Steroid glycoalk), ~~43 Vincamin (Alk)~~, 44 Cotyledon pilule (Control) (* occurring in *Fabaceae*). Crossed-out compounds are not represented at this concentration.

Genistein (an Isoflavonoid) (41) and Digitonin (a Cardenolide) (21) are most separated from the other compounds for the 5 w/w% concentration data. Nicotine H-tartrate* (an Alk) (37) and Solasodine (a Steroid glycoalk) (42) also stand out.

The cluster analysis showed a close grouping of the compounds. At a similarity of 0.90, there are four singletons (L-canavanine (Nonprotaa), Tannin (Polyphenol), Digitonin (Cardenolide) and Genistein (Isoflavonoid)), a cluster of two compounds (Nicotine H-tartrate (Alk) and Tropinone (Alk)) and the other 29 compounds in a single cluster.


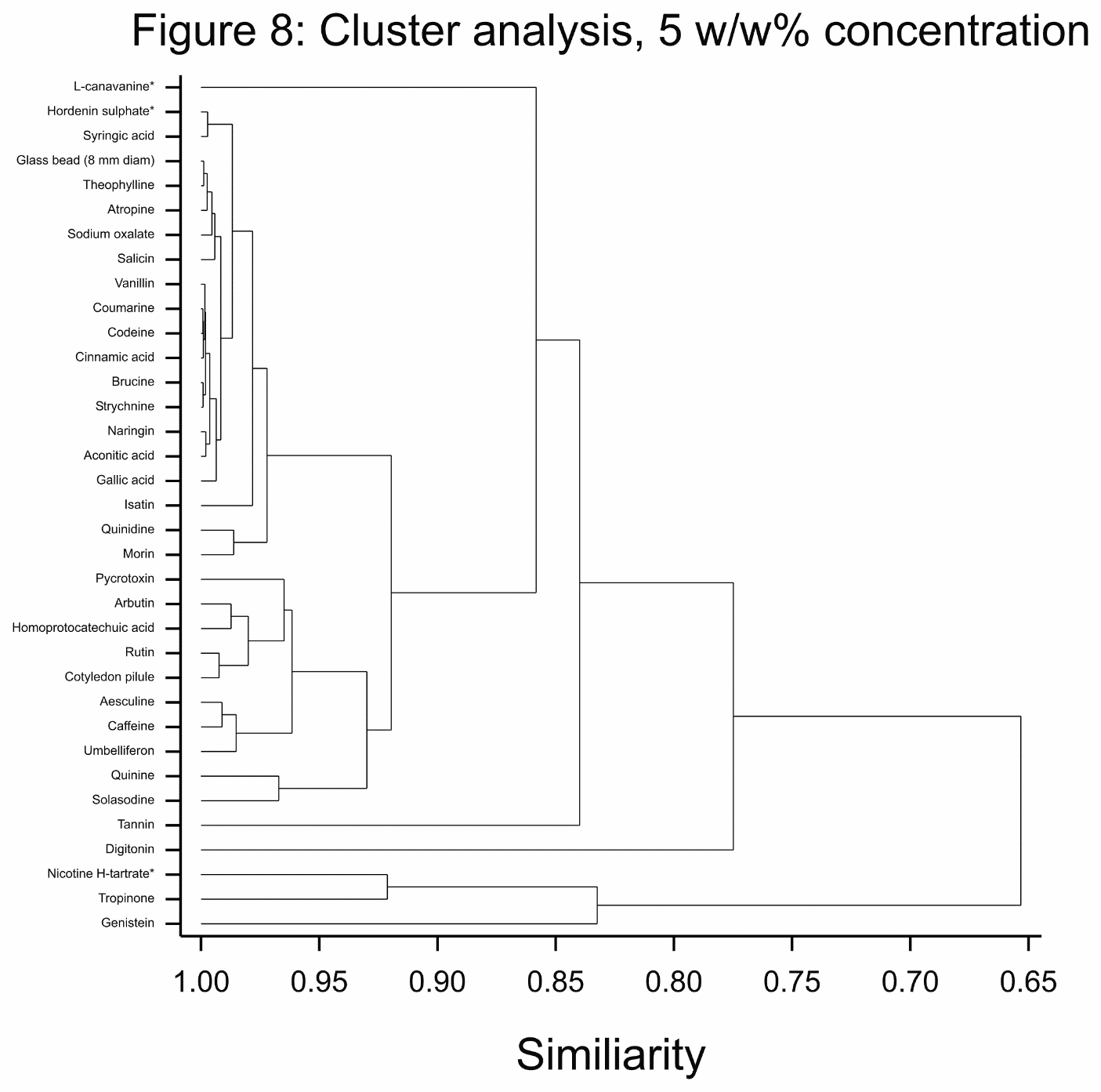


**Appendix: Raw Means and SEs**

**Concentration 0.0 0.1**

**Mean SEM Mean SEM**

**Compound**

Cotyledon pilule 16.72 2.770

Glass bead 9.50 2.127

L-canavanine 7.92 2.360

Hordenin sulphate 16.83 6.741

Condensed tannin 9.67 2.867

Tannin 14.00 3.570

Pycrotoxin 11.08 3.648

Quinidine 6.42 2.224

Syringic acid 12.08 4.693

Smilagenine 12.67 2.934

Vanillin 4.17 1.308

Theophylline 6.67 2.112

Naringin 9.33 5.262

Eserine 15.75 6.459

Morin 8.25 1.643

Gallic acid 11.67 3.744

Coumarine 0.25 0.179

Arbutin 11.58 3.815

Homoprotocatechuic acid 12.17 2.335

Aesculine 14.25 3.201

Caffeine 7.08 2.330

Digitonin 12.67 3.644

Barbitural 8.25 1.863

Reserpine 7.67 1.597

Hecogenin 11.75 2.944

Cinnamic acid 12.75 3.477

Umbelliferon 14.67 5.266

Tomatine 6.00 2.004

Rutin 7.58 1.990

Salicin 10.58 3.064

Isatin 15.08 3.276

Brucine 10.50 5.297

Strychnine 3.50 1.384

Quinine 9.67 2.698

Ergotamin 11.67 3.811

Codeine 6.33 1.082

Aconitic acid 11.25 3.861

Nicotine H-tartrate 10.92 4.610

Sodium oxalate 4.33 1.427

Tropinone 11.67 3.604

Atropine 13.25 4.918

Genistein 16.08 6.783

Solasodine 9.75 2.761

Vincamin 7.75 2.632

**Concentration 1.0 5.0**

**Mean SEM Mean SEM**

**Compound**

L-canavanine 23.00 7.138 32.83 11.105

Hordenin sulphate 11.75 3.262 10.75 3.631

Condensed tannin 13.33 3.120

Tannin 15.00 4.312 43.17 11.646

Pycrotoxin 8.17 2.561 18.25 4.755

Quinidine 8.33 2.039 11.08 3.151

Syringic acid 8.58 1.703 12.67 4.004

Smilagenine 47.42 8.809

Vanillin 0.50 0.289 2.08 1.234

Theophylline 11.17 3.191 9.50 2.479

Naringin 6.50 2.054 3.92 1.090

Eserine

Morin 7.00 1.303 14.33 5.974

Gallic acid 3.67 1.563 6.42 3.343

Coumarine 0.00 0.000 0.00 0.000

Arbutin 18.58 7.274 25.17 4.800

Homoprotocatechuic acid 29.50 12.235 29.58 3.185

Aesculine 4.08 1.221 34.42 7.742

Caffeine 9.25 2.042 26.75 4.927

Digitonin 9.92 2.661 46.92 15.405

Barbitural 13.25 6.401

Reserpine 9.50 2.843

Hecogenin 12.42 2.893

Cinnamic acid 4.08 1.252 1.75 0.845

Umbelliferon 20.25 5.836 35.92 7.310

Tomatine 9.67 3.315

Rutin 18.33 5.102 35.67 7.553

Salicin 5.50 1.991 9.92 2.762

Isatin 21.00 4.232 16.17 5.016

Brucine 0.92 0.379 3.00 1.279

Strychnine 2.17 0.588 4.08 1.288

Quinine 12.58 3.269 17.83 5.915

Ergotamin

Codeine 1.92 0.633 1.50 0.723

Aconitic acid 18.08 4.834 6.17 1.874

Nicotine H-tartrate 33.58 9.494 53.58 14.304

Sodium oxalate 6.00 2.157 10.42 2.838

Tropinone 34.42 5.206 60.75 6.320

Atropine 8.33 3.941 7.92 1.769

Genistein 15.67 7.398 86.08 15.035

Solasodine 30.25 10.878

Vincamin 4.75 1.533
