# Supplementary material for: A semiochemical view of the ecology of the seed beetle Acanthoscelides obtectus Say (Coleoptera: Chrysomelidae, Bruchinae)
Source: Ann Appl Biol. 2023 Sep 4;184(1):19–36. doi: 10.1111/aab.12862 (PMC10953445; doi:10.1111/aab.12862)
Supplement: Supplementary file 6 — Data S6. Supporting information. [file AAB-184-19-s008.docx]

A semiochemical view of the ecology of the seed beetle *Acanthoscelides obtectus* Say (Coleoptera: Chrysomelidae, Bruchinae)

József Vuts, Stephen J Powers, Eudri Venter, Árpád Szentesi

**The effect of secondary plant substances (SPSs) on bean beetle egg laying**

**Data and Methods**

A total of 37 compounds in three different classes were to be compared in binary choice tests. The compounds (with chemical formula) by class, corresponding control treatment and set within which experimentation was done, were:

**Class Compound Control Set**

Organic acids Oxalic acid (C2H2O4.2H2O) Distilled water 1

Nicotinic acid (C6H5O2N) Distilled water 1

cis-Aconitic acid (C6H6O6) Distilled water 1

Tartaric acid (C4H6O6) Distilled water 2

Fumaric acid (C4H4O4) Distilled water 2

DL-Malic acid (C4H6O5) Distilled water 2

Salicilic acid (C7H6O3) EtOH 3

Maleic acid (C4H4O4) EtOH 3

Succinic acid (C4H6O4) Distilled water 3

Malonic acid (C3H4O4) Distilled water 2

Sodium-citrate (C6H5O7Na3.2H2O) Distilled water 2

Magnesium citrate (Mg3(C6H5O7)2.H2OA) Starch solution 4

Others Rutin (Quercetin-3beta-rutinoside) EtOH 1

Codeine (C18H21O3N.H2O) EtOH 1

Salicin (C13H18O7) Distilled water 1

**Colchicine/0.004 M** (C22H25O6N) EtOH 5

**Ergotamine tartrate/0.007 M** (C33H35O5N5)2.(C4H6O6) EtOH 5

**Tomatine/0.086 M** (C50H83NO2) EtOH 6

Morin (C15H10O7) EtOH 6

Brucine (C23H26N2O4) EtOH 6

Isatin (C8H5NO2) EtOH 6

**Tannin (catechin)/2% w/v** EtOH 6

Solasodine EtOH 6

Atropine (C17H23O3N) EtOH 4

Nicotine H-tartrate (C14H18O5N2) Distilled water 4

Carbohydrates D-Raffinose (C18H32O16.5H2O) MetOH 5

L-Rhamnose (C6H12O5.H2O) Distilled water 5

Galactose (C6H12O6) Distilled water 5

D-Mannosen (C6H12O6) Distilled water 5

Saccharose (C12H22O11) Distilled water 2

D-Glucose (C6H12O6) Distilled water 3

D-Sorbit (C6H14O6) Distilled water 3

L-Arabinose (C6H10O5) Distilled water 4

D-Arabinose (C6H10O5) Distilled water 4

D-Fructose (C6H12O6) Distilled water 4

D-Xylose (C6H10O5) Distilled water 4

**Dextran/0.01 M** (H(C6H10O5)×OH) Distilled water 7

The concentration used was 0.1 M unless as stated for compounds in bold above.

A given compound and its corresponding control were compared in a 10 cm diam. glass Petri-dish divided into four sections by a paper or glass cross stuck to the bottom to hinder mixing of treated and control beans. The same treatment was placed at the opposite sections of the dish and the orientation of dishes was randomised. A mass of 32 g beans (var. Közép fehér) could be put into a Petri-dish, so ca. 8 g of either control or treated beans were spread into each section. Then, 10 male and 10 female beetles, 2-3 days old, were placed in a dish for 10 days with the dish being kept at 23 C in complete darkness. For each comparison, seven, or nine (for Atropine, Nicotine H-tartrate, L-Aribinose, D-Aribinose, D-Fructose and D-Xylose) replicate dishes were used. The replicates for each compound were necessarily all done together upon preparation of the compound, but due to the large number of compounds to be trialled, these were grouped into sets to be run at the same time. The allocation to sets is given in the table above.

After 10 days, the number of eggs laid into respective sections was counted. These numbers were used to calculate a per Petri dish index for eggs laid:

Egg Index = 100 × (No. of eggs laid on control – No. eggs laid on treated)/Total no. of eggs laid

This it gives a value between -100 and +100% for analysis.

For each compound separately having summed over the two sections per treatment within each Petri dish, a paired t-test was applied to the counts to obtain the overall significance of the effect on egg laying. A one-sample t-test was also applied to the egg index data, testing for a significant difference from 0% for the mean of the sample.

The complete set of data was also analysed using a linear mixed model, accounting for the design characteristics (random effects) of sets of compounds being assayed at different times, Petri dishes and sections within Petri dishes as a split-plot structure. The treatment term (fixed effects) in the model is then the factor of all the compounds and their corresponding controls. Although this is not an ideal situation for making comparisons of compounds *per se*, due to the imbalance, *i.e.* with compounds not being seen in the same sets nor together in Petri dishes, nor being all paired with the *same* control, the analysis still allows for tentative assessment, based on the underlying variance of the full set of data. The model was:

*Log*(*Count* + 0.5) = *Constant* + *Class*/*Treatment* + *Set*/*Dish*/*Section*

The Genstat (19^th^ edition, VSN International Ltd, Hemel Hempstead, UK) statistical package was used for this analysis.

**Results**

**Paired t-test results**

The table shows the results of the paired t-tests.

| **Class and Compound** | **Control mean** | **Treated mean** | **Mean of differences** | **SE** | **t-statistic** | **df** | **p-value** |
| --- | --- | --- | --- | --- | --- | --- | --- |
| **Organic Acids** | | | | | | | |
| Oxalic acid | 310.7 | 76.1 | 234.6 | 34.78 | 6.74 | 6 | <0.001 |
| Nicotinic acid | 415.1 | 62.7 | 352.4 | 32.10 | 10.98 | 6 | <0.001 |
| **cis-Aconitic acid** | **443.0** | **48.0** | **395.0** | **24.07** | **16.41** | **6** | **<0.001** |
| Tartaric acid | 274.1 | 150.7 | 123.4 | 44.56 | 2.77 | 6 | 0.032 |
| Fumaric acid | 324.6 | 84.6 | 240.0 | 24.23 | 9.91 | 6 | <0.001 |
| DL-Malic acid | 306.9 | 129.9 | 177.0 | 33.44 | 5.29 | 6 | 0.002 |
| Salicilic acid | 309.6 | 98.7 | 210.9 | 41.28 | 5.11 | 6 | 0.002 |
| Maleic acid | 415.6 | 47.6 | 368.0 | 30.69 | 11.99 | 6 | <0.001 |
| Succinic acid | 395.1 | 101.3 | 293.9 | 21.51 | 13.66 | 6 | <0.001 |
| **Malonic acid** | **301.3** | **109.0** | **192.3** | **21.42** | **21.42** | **6** | **<0.001** |
| Sodium-citrate | 249.1 | 238.6 | 10.57 | 45.14 | 0.23 | 6 | 0.823 |
| Magnesium citrate | 286.9 | 141.6 | 145.3 | 19.05 | 7.63 | 8 | <0.001 |
| **Others** | | | | | | | |
| **Rutin** | **443.1** | **32.4** | **410.7** | **14.16** | **29.00** | **6** | **<0.001** |
| Codeine | 380.4 | 81.3 | 299.1 | 36.35 | 8.23 | 6 | <0.001 |
| Salicin | 326.4 | 102.6 | 223.9 | 17.78 | 12.59 | 6 | <0.001 |
| Colchicine/0.004 M | 314.0 | 182.9 | 131.1 | 39.88 | 3.29 | 6 | 0.017 |
| Ergotamine tartrate/0.007 M | 302.7 | 140.4 | 162.3 | 20.38 | 7.96 | 6 | <0.001 |
| Tomatine/0.086 M | 337.6 | 102.9 | 234.7 | 18.64 | 12.59 | 6 | <0.001 |
| **Morin** | **420.1** | **17.6** | **402.6** | **12.46** | **32.31** | **6** | **<0.001** |
| **Brucine** | **380.4** | **20.0** | **360.4** | **11.77** | **30.61** | **6** | **<0.001** |
| Isatin | 368.6 | 70.7 | 297.9 | 24.11 | 12.36 | 6 | <0.001 |
| **Tannin (catechin)/2% w/v** | **403.6** | **31.7** | **371.9** | **10.19** | **36.49** | **6** | **<0.001** |
| Solasodine | 363.3 | 47.6 | 315.7 | 29.68 | 10.64 | 6 | <0.001 |
| Atropine | 380.8 | 51.4 | 329.3 | 21.54 | 15.29 | 8 | <0.001 |
| NicotineH tartrate | 295.2 | 144.8 | 150.4 | 25.11 | 5.99 | 8 | <0.001 |
| **Carbohydrates** | | | | | | | |
| **D-Raffinose** | **244.6** | **198.0** | **46.57** | **16.66** | **2.80** | **6** | **0.031** |
| L-Rhamnose | 275.6 | 242.7 | 32.86 | 24.70 | 1.33 | 6 | 0.232 |
| Galactose | 249.3 | 217.7 | 31.57 | 18.42 | 1.71 | 6 | 0.137 |
| D-Mannose | 259.1 | 262.1 | -3.00 | 19.06 | -0.16 | 6 | 0.880 |
| Saccharose | 277.6 | 210.4 | 67.14 | 31.37 | 2.14 | 6 | 0.076 |
| D-Glucose | 271.3 | 222.0 | 49.29 | 30.09 | 1.64 | 6 | 0.153 |
| **D-Sorbit** | **279.3** | **143.7** | **135.6** | **20.72** | **6.54** | **6** | **<0.001** |
| L-Arabinose | 228.8 | 193.2 | 35.56 | 21.07 | 1.69 | 8 | 0.130 |
| D-Arabinose | 240.1 | 203.4 | 36.67 | 23.94 | 1.53 | 8 | 0.164 |
| **D-Fructose** | **281.1** | **165.9** | **115.2** | **21.41** | **5.38** | **8** | **<0.001** |
| D-Xylose | 223.0 | 211.1 | 11.89 | 16.53 | 0.72 | 8 | 0.492 |
| **Dextran/0.01 M** | **280.6** | **66.6** | **214.0** | **19.37** | **11.05** | **8** | **<0.001** |

The results for the organic acids show that malonic acid and cis-Aconitic acid gave the most significant (p <0.001, paired t-test) results, having the greatest t-statistics. In particular, beetles were attracted to lay most eggs on cis-Aconitic acid-treated beans, compared to the other organic acids. Nicotinic acid, Maleic acid and Succinic acid also gave highly significant (p < 0.001, paired t-test) results with the next greatest mean of differences. For the other compounds, Tannin, Morin, Brucine and Rutin gave impressive results, with Tannin having the greatest t-value. There were only four statistically significant (p < 0.05, paired t-test) results for the carbohydrates (D-Raffinose, D-Sorbit, D-Fructose and Dextran) with Dextran providing the most statistically significant (p < 0.001, paired t-test) result with the greatest mean of differences.

The table below shows that the one-sample t-tests on egg index data gave results similar to those for the paired t-tests on count data, albeit with Maleic acid rather than Malonic acid being most significant (p < 0.001, t-test) along with cis-Aconitic acid for the organic acids, having accounted for total eggs laid.

| **Class and Compound** | **Mean of Egg Index** | **SE** | **t-statistic** | **df** | **p-value** |
| --- | --- | --- | --- | --- | --- |
| **Organic acids** | | | | | |
| Oxalic acid | 59.70 | 7.646 | 7.81 | 6 | <0.001 |
| Nicotinic acid | 73.24 | 3.924 | 18.67 | 6 | <0.001 |
| **cis-Aconitic acid** | **80.34** | **2.672** | **30.07** | **6** | **<0.001** |
| Tartaric acid | 27.82 | 9.899 | 2.81 | 6 | 0.031 |
| Fumaric acid | 58.08 | 4.736 | 12.26 | 6 | <0.001 |
| DL-Malic acid | 39.98 | 7.103 | 5.63 | 6 | 0.001 |
| Salicilic acid | 48.60 | 9.884 | 4.92 | 6 | 0.003 |
| **Maleic acid** | **78.88** | **3.399** | **23.21** | **6** | **<0.001** |
| Succinic acid | 59.33 | 4.370 | 13.58 | 6 | <0.001 |
| Malonic acid | 46.52 | 3.843 | 12.11 | 6 | <0.001 |
| Sodium-citrate | 1.970 | 9.795 | 0.20 | 6 | 0.847 |
| Magnesium citrate | 34.37 | 4.952 | 6.94 | 8 | <0.001 |
| **Others** | | | | | |
| **Rutin** | **86.33** | **1.993** | **43.33** | **6** | **<0.001** |
| Codeine | 63.74 | 4.483 | 14.22 | 6 | <0.001 |
| Salicin | 52.86 | 4.882 | 10.83 | 6 | <0.001 |
| Colchicine/0.004 M | 26.10 | 7.902 | 3.30 | 6 | 0.016 |
| Ergotamine tartrate/0.007 M | 38.31 | 5.843 | 6.56 | 6 | <0.001 |
| Tomatine/0.086 M | 54.06 | 3.740 | 14.46 | 6 | <0.001 |
| **Morin** | **91.90** | **1.060** | **86.68** | **6** | **<0.001** |
| **Brucine** | **89.98** | **1.999** | **45.02** | **6** | **<0.001** |
| Isatin | 67.30 | 3.715 | 18.12 | 6 | <0.001 |
| **Tannin (catechin)/2% w/v** | **85.68** | **2.829** | **30.29** | **6** | **<0.001** |
| Solasodine | 75.89 | 3.723 | 20.39 | 6 | <0.001 |
| Atropine | 76.70 | 4.733 | 16.21 | 6 | <0.001 |
| NicotineH tartrate | 35.40 | 6.024 | 5.88 | 8 | <0.001 |
| **Carbohydrates** | | | | | |
| **D-Raffinose** | **11.00** | **4.035** | **2.73** | **6** | **0.034** |
| L-Rhamnose | 6.00 | 4.813 | 1.25 | 6 | 0.259 |
| Galactose | 7.31 | 4.205 | 1.74 | 6 | 0.133 |
| D-Mannose | -0.50 | 3.757 | -0.13 | 6 | 0.899 |
| Saccharose | 14.51 | 6.459 | 2.25 | 6 | 0.066 |
| D-Glucose | 11.25 | 6.051 | 1.86 | 6 | 0.112 |
| **D-Sorbit** | **32.13** | **4.680** | **6.86** | **6** | **<0.001** |
| L-Arabinose | 7.85 | 5.052 | 1.55 | 8 | 0.159 |
| D-Arabinose | 8.11 | 5.133 | 1.58 | 8 | 0.153 |
| **D-Fructose** | **26.58** | **5.367** | **4.95** | **8** | **0.001** |
| D-Xylose | 2.86 | 4.04 | 0.71 | 8 | 0.499 |
| **Dextran/0.01 M** | **61.54** | **5.169** | **11.90** | **8** | **<0.001** |
