# Supplementary material for: A semiochemical view of the ecology of the seed beetle Acanthoscelides obtectus Say (Coleoptera: Chrysomelidae, Bruchinae)
Source: Ann Appl Biol. 2023 Sep 4;184(1):19–36. doi: 10.1111/aab.12862 (PMC10953445; doi:10.1111/aab.12862)
Supplement: Supplementary file 7 — Data S7. Supporting information. [file AAB-184-19-s004.docx]

A semiochemical view of the ecology of the seed beetle *Acanthoscelides obtectus* Say (Coleoptera: Chrysomelidae, Bruchinae)

József Vuts, Stephen J Powers, Eudri Venter, Árpád Szentesi

**The effect of different secondary plant substances (SPSs) on fecundity**

**Data and Methods**

**Response to concentration of tannin study**

Seven concentrations of tannin (0.0004, 0.0013, 0.004, 0.012, 0.037, 0.111 and 0.33 M) and a control (EtOH-treated seeds) were set up. Three treated or control beans were placed in a 5 cm high × 2 cm diam. vial with three females and three males, 3-4 days old. There were 39 replicate vials per treatment. All replicates and treatments were independent. All concentrations and hence the full set of 312 vials were set up and the experiment started on the same day. It lasted for six days with the vials being kept in complete darkness at 23 C. Vials of each concentration were placed on separate trays but the position of trays in the controlled environment, and the position of vials on trays was completely randomised for the experimental design. After the six days, the number of eggs/vial was counted.

Analysis of Variance (ANOVA) was applied to the data to compare the effect of the concentrations overall. No transformation of the data was required, the residual plots showing that the data on the raw scale satisfied the assumptions of the analysis.

A logistic curve model was used to describe the pattern of decreasing eggs laid with increasing concentration. The model was:

*Eggs* = *C*/(1 + *exp*[*B*log_e_(Concentration) – *M*])

where *B* is a measure of the increasing response caused by increasing concentration (i.e. the “slope” of the curve), *M* is the log_e_(Concentration) which reduces the number of eggs laid by 50% (the ED50) and *C* is the control response. The method of nonlinear least squares regression was used to fit the model and estimate the parameters *B*, *C* and *M* with standard errors.

The Genstat (19^th^ edition, VSN International Ltd, Hemel Hempstead, UK) statistical package was used for the analysis.

**Results**

There was a highly significant (p < 0.001, F-test) effect of concentration of tannin.

Analysis of variance

====================

Variate: Eggs

Source of variation d.f. s.s. m.s. v.r. F pr.

Conc 7 433466.4 61923.8 94.03 <.001

Residual 304 200207.9 658.6

Total 311 633674.3

The means were:

Conc 0.0000 0.0004 0.0013 0.0040 0.0120 0.0370 0.1110

113.2 95.5 93.4 95.8 51.2 30.1 19.6

Conc 0.3300

13.6

n = 39; SED = 5.81 on 304 df; LSD (5%) = 11.44

The means show that a concentration greater than 0.004 M is required for there to be a major response to tannin, with doses greater than 0.012 M being increasingly inhibitive to egg laying.

The logistic model gave a reasonable fit to the data with R^2^ = 65.6% variance explained. The regression ANOVA and estimated parameters with SEs was:

Summary of analysis

-------------------

Source d.f. s.s. m.s. v.r.

Regression 3 1696730. 565576.7 806.04

Residual 309 216817. 701.7

Total 312 1913547. 6133.2

Percentage variance accounted for 65.6

Standard error of observations is estimated to be 26.5.

Estimates of parameters

-----------------------

Parameter estimate s.e.

B 0.8389 0.0899

C 109.46 3.68

M -4.237 0.158

The LD50 (*exp*(*M*)) was therefore 0.01441 (0.00229) M of tannin. The standard error of means given the model was 4.24, as plotted on the graph of the fitted model below.

**Figure 1.** Mean number of eggs laid with standard error, a fitted logistic curve relationship and the standard error of the mean number of eggs laid given the model (SEM). The estimated parameters (SE) in the model, *Eggs* = *C*/(1 + *exp*[*B*log_e_(Concentration) – *M*]), were *B*: 0.8389 (0.0899), *C*: 109.46 (3.68) and *M* -4.237 (0.158). The LD50 (*exp*(*M*)) was 0.01441 (0.00229). Variance explained (R^2^) was 65.6%.
